# Face mask is an efficient tool to fight the Covid-19 pandemic and some factors increase the probability of its adoption

**DOI:** 10.1038/s41598-023-34776-7

**Published:** 2023-06-06

**Authors:** Olivier Damette, Toan Luu Duc Huynh

**Affiliations:** 1 BETA, University of Lorraine, France and CEC Paris Dauphine, Paris, France; 2 University of Economics Ho Chi Minh City, Ho Chi Minh City, Vietnam

**Keywords:** Infectious diseases, Health policy, Public health, Epidemiology, Human behaviour

## Abstract

This study examines the dynamic impact of face mask use on both infected cases and fatalities at a global scale by using a rich set of panel data econometrics. An increase of 100% of the proportion of people declaring wearing a mask (multiply by two) over the studied period lead to a reduction of around 12 and 13.5% of the number of Covid-19 infected cases (per capita) after 7 and 14 days respectively. The delay of action varies from around 7 days to 28 days concerning infected cases but is more longer concerning fatalities. Our results hold when using the rigorous controlling approach. We also document the increasing adoption of mask use over time and the drivers of mask adoption. In addition, population density and pollution levels are significant determinants of heterogeneity regarding mask adoption across countries, while altruism, trust in government and demographics are not. However, individualism index is negatively correlated with mask adoption. Finally, strict government policies against Covid-19 have a strong significant effect on mask use.

## Introduction

To confront the global Covid-19 pandemic and reduce the spread of the virus, we need to improve our understanding of the factors that influence its spread. Many governments and public health departments have promoted and imposed various mitigation measures to contain the spread of Covid-19^3^ and^7^; indeed, such measures were crucial when no vaccine were available and are still important since herd immunity with vaccination is far to be achieved. The heterogeneity in fatality rates across the globe reflects differences in how well countries have managed the pandemic^49^, the effectiveness of the various policies, and the extent to which they have been promoted by public authorities and adopted by populations.

One of the most widely debated of such policies, especially among the general public, is the wearing of face masks. While face mask use is encouraged by most governments, support for their use has been limited among general populations^90^. The effectiveness of masks in reducing the transmission of Covid-19 has been contested, and levels of self-reported mask use differ considerably between countries^34^. We also acknowledge the existing literature of Lu et al.^58^ in terms of collectivism and mask-wearing behavior. Interestingly, the national data within the US could be aligned with our international scope. According to a study by English et al.^27^, almost everyone in China (up to 94%) wore masks during the first 14 days of the outbreak, which could be a reason why the number of cases in China decreased significantly 14-21 days later. The authors also discovered that higher levels of air pollution in the past were linked to quicker adoption of mask usage. This research supports the authors’ argument that there is a connection between air pollution and the use of masks. Although there are some existing empirical findings about cultural dimensions (collectivism), environmental conditions, and protective behaviors, these studies and this paper converged similar findings. Our study differs from these works based on the data scope. While the current work only focuses on a single country, our study expands the results internationally.

To the best of our knowledge, we present here the first statistical analysis of the effectiveness of mask-wearing to reduce the spread of Covid-19 at a global scale. 1) We compute dynamic panel econometric estimates to assess the impact of mask-wearing on both infections and fatality rates per capita. Although some data is available on the number of tests being conducted, no official data on mask-wearing exists. Previous studies used a dummy variable by considering the date of mandatory mask policy introduction and the duration variable^52^. For this study, we collected individual data from the Covid-19 World Survey Data API (v1.2) jointly conducted by the University of Maryland and Facebook^6,29,50^. We obtained estimates of the percentage of people in a given country that used face masks daily from April, 23 to July, 15, 2020. Concerning this last point, the use of Kreuter et al. study^50^ from the University of Maryland also proved the tremendous potential of using social media platforms to obtain quick, large-scale population response and attitude toward social policy. By employing the available data from April, 23 to July, 15, 2020, implying the previous period before pharmaceutical intervention, we mainly focus on the first wave of the pandemic but after the peak of the number of Covid-19 cases and main lockdown policies in most of OECD countries took place. Our sample is in the vein of previous literature: Lyu and Wehby^59^ used daily data between March 31 and May 22, 2020 while Chernozhukov et al.^15^ used a dataset from March 7, 2020 to June 3, 2020. Two main other reasons might explain why this period should be taken into account. First, when the outbreak of the COVID-19 pandemic happened, there is an ambiguity in containing the spread of this deadly virus. People started wearing masks to protect themselves against the airborne despite having a controversial decision. We can observe that this action in the first wave is the only measure that people can deal with. Second, in the next waves, the government might carry several interventions such as severe lockdown, vaccinations, social distancing, and so forth. Thus, it is pretty challenging to disentangle the mask-wearing effect from other interventions in complicated interactions. Moreover, people are likely to update their beliefs after receiving much information regarding the COVID-19 pandemic, implying the human behavior changes in the next waves. Therefore, focusing on the first and foremost period could offer insights into the nature of determinants of wearing masks and their effectiveness. We developed a dynamic model that included lagged effects to control the epidemic’s dynamics over time, including delays between infection and case confirmation and incubation period. Our dynamic model indirectly accounts for potential simultaneous impacts of other determinants of the Covid-19 outbreak. We also included additional controls to directly account for certain factors that may have influenced viral transmission (e.g., mobility, temperatures). Finally, to preserve our causal identification from reverse causality bias, we controlled for the effects of other non-pharmaceutical mitigation measures during the studied period by adding the number of Covid-19 tests (testing policy) and a stringency index (reflecting all mitigation measures) as additional explanatory variables. 2) We then performed a cross-sectional statistical analysis to examine the socio-economic determinants of mask-wearing across countries. For this analysis, we examined a different set of determinants to understand the heterogeneities regarding mask adoption across countries and the factors that can increase the number of individuals that wear a face mask.

This paper contributes to the existing literature on two main pillars. First, this paper looks at the effects of wearing a mask on COVID-19 morbidity and mortality before having any pharmaceutical intervention such as vaccination or medical treatment^69^. Second, this study also explores the determinants of mask use, such as socio-economic factors, culture, population, etc. By doing that, we can disentangle the heterogeneity among countries since our study looks at the global database. More importantly, to deal with the problems during the pandemic, we need to understand the local (or country) context to generalize the global context, which suggests better public administration and governance.

Section “Data and methods” lays out our data and econometric methodology. Section “Panel data and sample issues” discusses our two main findings, such as (i) the effectiveness of wearing masks and (ii) determinants of wearing masks on a global scale with different models and approaches to ensure robust findings. Finally, Sect. “Definitions of variables and data sources” concludes.

## Literature review

This absence of consensus among general populations can be explained not only by differences in individuals’ subjective concerns (Perotta et al., 2020) but also by conflicting national guidelines and public communication. The latter is a crucial area of concern since political leaders’ words and actions affect people’s behavior^1^; people need official guidance^54^. However, public guidance has been subject to much fluctuation and is characterized by a lack of consistency among political leaders. President Trump in the US is a prime example of a leader whose opinions on this issue have been inconsistent. Likewise, the French government did not support face mask use last March 2020, considering it to be ineffective, but changed its guidance to support the wearing of face masks in late May. Even the World Health Organization initially advised against widespread mask wearing before changing its recommendation on June, 5, 2020 (Lefler et al. (2020), WHO^91^). Beyond individual-level variations, heterogeneity between countries still exists; mask wearing has been recommended since the early stages of the pandemic by governments in East and South East Asia. It was only later recommended in many Western countries and is still not recommended at all in some Nordic countries. Policies on face mask use are also more strictly enforced in Asian countries. The rationale choice for this is that a voluntary policy would likely lead to insufficient compliance and would, hence, be less effective than a mandatory policy^9^.

More noticeably, the literature on determinants of wearing masks is still ongoing. Since the COVID-19 pandemic is known as the novel deadly virus without clear, responsive policies, different countries might follow their strategies, which leads to the divergence of health policies. Motivated by the partisan differences in Americans, the current empirical studies come into the agreement that there is a disproportionate correlation of partisanship and behavioral responses to the COVID-19 pandemic, such as social distancing^2^, mask-wearing^63^, and even the political voting^4^. An inconsistent public message could drive people’s attention to this deadly virus in different ways. Accordingly, President Trump and other Republican officials tended to underestimate the severity of COVID-19 in the beginning outbreak while the opposite side continually criticized and put more effort into sounding an alarm to the community. This case naturally exemplifies what happened in the United States while there is still a gap to understand for the worldwide scope.

However, the effectiveness of mask wearing policies to reduce the spread of the Covid-19 virus, especially when they go beyond the precautionary principle, has been the subject of much scientific debate (Perotta et al., 2020). Studies on the role face masks can play in mitigating the pandemic are scarce, particularly statistical and global-scale studies. This is partly due to limited data at the level of countries and the difficulty of conducting clinical trials (which may be regarded as exploiting vulnerable populations) at the individual level.

A very small body of literature has found that mandatory and voluntary mask policies should mitigate the transmission and, thus, the spread of the Covid-19 pandemic. The effects of face masks have been examined in a large survey^16^ and a meta-analysis^16^. Both studies concluded that face mask use reduces the transmission of infected droplets in both laboratory and clinical contexts. In addition, public mask use is most effective in reducing the transmission of the virus when compliance is high and when there is a high level of trust in politicians^5^. While clinical masks are the best protective solution, surgical and comparable cloth masks have also been shown to reduce transmission, albeit in a less comprehensive way^16^.

Experimental studies on animals confirm the effectiveness of mask use: protected animals were found to be less infected and sick than their mask-free neighbors^13^. Results based on mathematical models^83^ show that face mask use by the public could make a major contribution to reducing the impact of the Covid-19 pandemic. If masks were used in public all the time (not just from when symptoms first appear), the effective reproduction number could be reduced to less than one^83^. From a theoretical and mathematical perspective, face masks, even if they have only a limited protective effect, can reduce the total number of infections and deaths and delay the peak of the epidemic^90^.

A small number of recent statistical studies have confirmed these results. Based on linear correlation and projections exercises and using data from the US, Italy, and Wuhan city in China, one study concluded that wearing a face mask in public is the most effective means of preventing Covid-19 transmission^92^. However, potential biases, especially regarding the failure to control for other non-pharmaceutical policies, have been reported. Very recently, an econometric study demonstrated the positive results of introducing masks for employees in US states in April 2020^15^. Lyu and Wheby^59^ conducted a quasi natural experiment for fifteen US states and show a reduction in the daily COVID-19 growth rate by 0.9 to 2 percentage points over time. Recently, Karaivanov et al.^48^ confirm this result using Province-level data in Canada over January-July 2020 period. Likewise, in Germany, the introduction of mandatory mask wearing has reduced the growth rate of Covid-19 cases. The study from Mitze et al.^64^ exploits the fact that the obligation to wear face masks in public transport, shops, and workplaces was introduced much earlier in Jena area (on 6th April) than in all other regions in Germany (around 20 days later). Generally, these findings are in agreement with the assumptions of epidemiologists and virologists regarding the benefits of reducing virus particle transmission by wearing a face mask^22,54^.

Little information is available on a global scale. However, an analysis of the socio-economic determinants of Covid-19 mortality across countries demonstrated that a longer duration of mask wearing by the public was negatively associated with mortality^52^. In this study, the effect of mask wearing is proxied by the delay between the first infected case and a government recommendation on mask wearing by the public. This study finds that ’in countries with public policies and cultural norms supporting public mask-wearing, per-capita coronavirus mortality increased on average by just 15.8% each week, as compared with 62.1% each week in other countries’.

Concerning the global cross-country determinants that influence attitudes to face masks, a small number of studies have been conducted on the level of individuals in Germany based on survey data^77^ and Facebook Health Behavior Survey conducted in eight industrialized countries (Perotta et al., 2020). The first one found that worries about the current pandemic have the largest positive influence on mask wearing. Self-protection, protection of others, and perceptions of others’ judgment - especially for young people - have also been found to be significant drivers. Demographic factors (e.g., age, gender) were not found to be significant drivers. The aforementioned study found that self-reported mask use differed considerably across the eight countries considered. In contrast to previous findings, sex- and age-specific patterns about threat perception, confidence in the healthcare system, and the likelihood of adopting preventive behaviors were also documented^34^. Older individual-level studies on the severe acute respiratory syndrome (SARS) epidemic of 2003 in Hong Kong^85^ reported that women, people in the 50-59 age group, and married respondents were more likely to wear face masks. Some other studies of different but related nature analysed the practices about face masks/N95 respirators utilization in Poland^31^ whereas (Betsch et al.) investigated the social and behavioral consequences of mask policies face with the Covid-19 pandemic using 7000 German participants from April, 14 to May, 26, 2020. They reveal that mandatory policy is more effective than voluntary policy. The story is not only wearing the mask but also the appropriate usage^40^. Concomitantly, the single-country survey highlights that risk perception might correlate with the mask-wearing behaviors^41^.

Regarding culture and human behaviors, Gelfand et al.^32^ shed a new light on the relationship between cultural tightness and historical rice farming cultures. In a study conducted by Gelfand et al.^32^, it was discovered that societies with strong cultural norms and strict rules had fewer COVID cases by October 2020. The authors of this study suggest that it would be valuable to investigate the cultural tightness scores before the pandemic as another factor in the adoption of masks and the reduction of COVID cases. According to a recent study by Talhelm^84^, historical societies that engaged in rice farming were more focused on prevention and were better equipped to respond collectively to the COVID-19 outbreak. During the initial year of the pandemic, rice farming societies across the globe had fewer cases and lower mortality rates. This provides additional evidence to support the idea that cultural practices like mask-wearing can play a role in preventing the spread of COVID-19. Therefore, our study sheds further light on cultural differences in COVID-19 outcomes.

To summarize our literature review, the synthesis of the literature on COVID-19 and public health admitted that wearing a mask could slow the speed of the spreading of coronavirus^62^. Although the strand of literature is growing on different aspects (for example, the politics of mask-wearing in the study of Kahane^46^), our study has its value by exploring the role of an immediate solution by using masks to halt the deadly spread of coronavirus before any pharmaceutical intervention.

## Data and methods

### Panel data and sample issues

We conducted an original empirical work based on a 96 countries dataset between the first of January and the 15th of July 2020, covering the entire “first wave” of the pandemic. As mentioned earlier, we only focused on the first wave to minimize the biases, which might be raised from updating beliefs from human behavior. In addition, we can disentangle the interference with other government policies, vaccination, and non-pharmaceutical policies since these regulations did not happen intensively and interactively. For the first section, we obtain a panel with 96 countries and around 7359 observations. We collected (1) the number of confirmed COVID-19 cases and deaths for the countries in our sample from the European Center for Disease, Control and Prevention between 1st January 2020 and July, 15th 2020, (2) the estimated population in 2019 from the World Bank’s World Development Indicators database, and (3) the mask-wearing variable from the Kreuter et al. study from University of Maryland using the Facebook platform^50^.

Our mask wearing variable is based on the Covid-19 World Survey Data API^29^ and^50^. The surveys ask respondents how many people in their household are experiencing Covid-19-like symptoms, among other questions. These surveys are voluntary, and individual survey responses are held by University of Maryland and are shareable with other health researchers under a data use agreement. No individual survey responses are shared back to Facebook. Using this survey response data, we estimate the percentage of people in a given geographic region that use face mask cover. We use the smoothed weighted (two sets of sample respondents separately are used (CMU US and UMD global surveys)) percentage of survey respondents across an one week window that have reported use mask cover (see also https://covidmap.umd.edu/document/css_methods_brief.pdf for more details).

The descriptive statistics (average mask-wearing proportion, in SI Appendix) seem coherent with other sources from the literature. For example, data based on Health Metrics and Evaluation at the University of Washington in Seattle reported in the USA. While Fetzer et al.^30^ and Van Bavel et al.^86^ constructed weights that account for the differential sample size across countries, the mask-wearing variable approach is also consistent with the previous data processing. By doing this, the dataset can be eliminated the sampling bias from country differences.

The dimension of our panel (96 countries) has been constrained by data availability about our main interest ‘mask’ variable and a significant number of respondents to the mask adoption survey. The countries in our sample have not encountered the first wave of the pandemic quite in the same time and the responsiveness rate about the mask wearing variable can be different between high-income and development countries. Thus, we also used an alternative proxy for mask use as a robustnes check by computing a dummy variable taking 1 for periods since when the government of the country has instated a mask requirements policy, following Chernozhukov et al.^15^ and Leffler et al.^52^.

Considering only European countries as a robustness check enables us to focus on countries with high responsiveness levels about the mask wearing variable to maximize the quality of the available information for this variable of great interest in our study. We also estimate our model on non European Countries and Asian countries separately (in SI Appendix). In addition, we have to take into account the fact that we have a panel of 96 countries with an important heterogeneity concerning the take-off Covid-19 periods (time with the first infected people) and so different Covid-19 dynamics over time: the first wave of Covid-19 epidemic has started later in Brazil than in Italy. Considering only homogeneous European countries is therefore a mean to test the presence of sample bias. We will show that results reported on European countries in our database are completely in accordance with the global results conducted on the global 96 countries sample.

### Definitions of variables and data sources

The paper is divided into two parts. The first one deals with mask effectiveness using panel data, and the second one deals with mask adoption drivers using cross-section data. In the following subsection, we would like to depict our data features as well as the relevant literature for choosing these following determinants in our models.

*Regarding the panel data group*, there are seven main variables that we used to construct the panel data set to estimate the effectiveness of wearing masks on infected cases and deaths. To be more precise, we have *Casepop* and *Deathpop* presenting Covid-19 cases and deaths on a daily basis. In addition, $New\_tests$ and *Masks* demonstrate the daily number of new Covid-19 tests per thousand and the number of individuals reporting a mask use, respectively. Furthermore, *Stringency* was collected to perform as a proxy for responsive government policies with several sub-categories. More importantly, *Temperatures* and *Mobility* were employed to capture the daily average temperature in each region and changes in human mobility. The detailed description for each variable can be found in Appendix (section 1.1). The current literature also confirms that the stringent policies could predict the COVID-19 outcomes^76^, even the strengthened features of the political regime^47^. Additionally, there is a correlation between the number of tests and reported cases and deaths (per capita), implying a must to control the country’s policies to detect the infected cases^36,45^. More importantly, differences in average temperature could significantly correlate with the transition of the disease even in an empirical study^67^ or meta-analysis with 517 papers^61^. The lower the human mobility, the lower the COVID-19 cases. These effects can be explained by the fewer social interactions^14,68,89^. To sum up, based on the previous literature, our estimations are designed rigorously and consider the previously updated literature to maintain reliable findings.

*When it comes to the cross-sectional data*, we constructed our data at the country-level to cover the socio-economic determinants. To be more precise, we capture the population features (Population density - *Density*; the aging population - *Age*65), environmental degradation (*CO*2), chronic diseases (*Diabetic* and *Overweight*), economic and educational status (economic status as *GDP*, school enrollment as *School* or reading ability *PISA*), differences in cultural and behavioral dimensions (*Altruism* index, *Tolerance* index, $Risk\_aversion$ index, and political trust $Government\_confidence$). All variables’ descriptions can be found in Appendix 1.2. These determinants as mentioned above, were acknowledged in the extant literature. Accordingly, the effects of population characteristics, including density and aging, significantly correlate with the COVID-19 severity^10,65^. This relationship is very intuitive because the higher population density represents the greater proportion of people who gather in common places, which exposes the higher risk of transmission. In addition, Viscusi^87^ contributes empirical evidence that the percentage of elderly people could positively predict the COVID-19 mortality rate per million population after controlling other factors such as the ranking of the health system, population density, economic freedom index, etc. for 180 countries. This finding is also consistent with the existing literature since the elder, known as those who are older than 65 years old, should be classified into the vulnerable group in society^38,56^. Since our cross-sectional data looks at the mask adoption drivers, environmental problems, proxied by the carbon emission level, should be included in the model. However, no study directly explores the inter-relationship between pollutants, mask-wearing, and COVID-19, the preliminary results of Persico & Johnson^72^ highlight that environmental degradation could positively contribute to the severity to COVID-19 infections. Concomitantly, the theoretical framework of Gondim^33^ admitted that several countries had the previous experience to deal with epidemics of respiratory diseases because of wearing surgical masks in public (See more at A quick history of why Asians wear surgical masks in public.). Therefore, we would like to control the environmental situation in our cross-sectional data. It is also worth mentioning that the large-scale survey from 49,968 participants across 67 countries of Van Bavel et al.^86^ indicates that social identity and collective behavior could correlate with the non-pharmaceutical interventions. Motivated by this finding, we would like to acknowledge the role of socioeconomic status and behavioral factors in our models. Therefore, we further address why people wear masks from different perspectives with the diversity of control variables, even social and behavioral determinants^86^. SI Appendix (Table S22 and following one) shows more detail of our descriptive statistics for all variables. Overall, there is no perfect correlation among predictors, reflecting the characteristics of the worldwide COVID-19 situation.

To elaborate our motivation and rationale choices for these variables, this study attempts to extend the variables options from the English et al.^27^ with the time dimension. By using a single country context (China), the study emphasizes that the number of days that could reduce the spread of the virus is around 14 to 21 days. Therefore, this study reapplies the evidence regarding the specific days for mask effectiveness. More noticeably, the literature review study of Howard et al.^39^ pointed out the majority of determinants of mask-wearing effectiveness. Therefore, we put these factors into account the global scope.

### The econometric panel model to assess mask effectiveness

To empirically test the effect of mask adoption on Covid-19 outcomes, we use a dynamic panel econometric model as follows:

1 $$\begin{aligned} \begin{aligned} y_{i,t} = \alpha _{0} + \underbrace{\alpha _{1} y_{i,t-1} + \alpha _{2} y_{i,t-k}}_{\text {pandemic dynamic effect}} + \underbrace{ \alpha _{3} C_{i,t-p}}_{\text {lagged mask effect}} + \alpha _{4} X_{i,t-p} + \mu _{i} + \delta _{t} + \varepsilon _{i,t} \end{aligned} \end{aligned}$$

Where the subscripts *i* and *t* represent country index and periods (days) respectively. The dependent variable, $y_{i,t}$, can be the number of infected individuals (casespop) or deaths (deathpop) per capita (considering the population size) at time *t*. $C_{i,t-p}$ is a vector of variables depicting the effects of mask wearing in day $t-p$. A vector of controls $X_{i,t-p}$ is included to deal with omitted bias and endogeneity issues. Country-specific fixed effect, $\mu _i$, are included to control for time-invariant omitted-variable bias and $\varepsilon _{i,t}$ is the error term. $\delta _{t}$ is a deterministic time trend that controls the deterministic dynamics of the epidemics over the studied period and captures some unobserved information about the pandemic common to all countries such as learning effects (see^24^ about including time trend to capture time-variant unobservables in panel MG regressions). Hence, our model captures both deterministic dynamics (the ’natural cycle’ of the virus) via deterministic trends and stochastic dynamics via the AutoRegressive structure. In addition, lagged terms $y_{i,t-k}$ capture the stochastic part of the pandemic dynamics.

Considering lags in epidemic dynamics is crucial on an theoretical/epidemiological point of view but also for causal identification concerns. Previous literature (Karaivanov20, Greenhalg et al. (2020)) have identified that threat perceptions about the current crisis can change the mask adoption and so human behaviors leading to potential reverse causality concerns (i.e. the concern that causality flows from COVID-19 to face mask use rather than from face mask use to COVID-19). Including lagged values of the explanatory variable in our model is a mean to deal with potential reverse causality issues. However, the use of lagged explanatory variables is sometimes insufficient to create the exogeneity necessary for causal claims as pointed out by Bellemare, Masaki, and Pepinsky^8^ or Leszczensky and Wolbring^53^.

In addition, there is a theoretical rationale for including lagged values of the independent variables in our model considering the epidemiological literature. In line with Chernozhukov et al.^15^ and Damette et al.^21^, we assume that the Covid-19 policies and behaviors affect cases and deaths with time lags: two weeks between changes in behavior or policies, and changes in reported cases are considered. Indeed, we assume *k* equal to 7 or 14 lags/days in our baselines specifications. This delay, in the way of Chernozhukov et al. (2020), is related to the existence of incubation (the time from infection to symptom onset) and confirmation periods (the time between symptoms or/and infection and infection and when a person is tested and appears in our data). Siordia^81^ show that the mean incubation time is estimated to around 3-9 days (see also Lauer et al.^51^) whereas Cereda et al.^12^ estimated an average of 7.3 days between symptom onset and reporting (a range between 1 to 20 days is considered in the literature) and Linton et al.^57^ and Sanche et al.^80^ estimated an average of 15-16 days from onset to fatality.

Moreover, for logical reasons, since the mask wearing do not immediately impact the Covid-19 spread, the mask wearing variables are also included in our model with a lag of order *p*. Indeed, there are delays between the time of potential avoided infection corresponding to mask wearing and the time of official counting of a potential infected individuals (or fatality). Therefore, when dealing with *p*, a minimum of 7 to 14 days is considered. A benchmark 28 lags (about one month) delay is considered when dealing with *deathpop* because of the more important lag length assumed between the infection and the deaths related to the Covid-19 virus. More longer delays of 42 and 56 days have been also considered in robustness checks to take into account the dynamic persistence of the pandemic (see also SI Appendix), the delay between the infection time and potential health problems and potential data reporting bias. *casepop* and *deathpop* respectively give a short-run and medium/long-run time perspective of the dramatic outcomes of the pandemic. Note that when $y_{i,t}$ is the fatality rate, we also add the ratio of infected cases per capita in our benchmark specification in order to account for the fact that the level of the pandemic can impact the fatality rate. The reason behind is to control for a level effect and a kind of saturation effect of the health system (too many infected individuals to manage is likely to finally increase the fatality rate).

### Identification issues and endogeneity

Equation (1) can be estimated by the mean group (MG) estimator introduced by^73^. Both estimators are relevant for macro panels such as the one used in this paper: *T* is equal to 84 and is thus close to $N=96$ (see SI Appendix). The Mean Group (MG) estimator consists in estimating each regression separately for each panel member *i* (country here) with a minimum of restrictions. All estimated coefficients are heterogeneous and are subsequently averaged across countries *via* a simple unweighted average (Pesaran and Smith, 1995; Eberhard and Presbitero, 2015; Samargandi et al., 2020). An intercept is included to capture country fixed effects as well as a linear trend. In the dynamic fixed effects (DFE), the slopes are homogeneous but the intercepts are allowed to vary across countries.

Although we apply appropriate macro-panel estimators to our data, several issues can nonetheless emerge. First, lot of dynamic models are vulnerable to the so-called Nickel bias. Here, this bias is very negligible, notably considering the important time length of our series. Second, panel regressions may be exposed to an omitted variables bias. It would be possible to include control variables such as other control measures (e.g. testing, travel controls) or structural determinants (e.g. population density and demographics such as the population over 65, tourists flows, GDP per capita, and measures of health infrastructures). Considering the so-called problem of ’bad controls’, our set of explanatory variables is assumed to be restricted to the mask variable in order to avoid an over-controlling problem^23^. In addition, considering data availability and the fact they are time-invariant variables, we capture these unobservables *via* the lagged term $y_{i,t-1}$ and above all with country fixed effects. Another identification issue is related to the potential reverse causality bias related to our Covid-19 variables: news about contemporaneous dynamics of the Covid-19 outbreaks and counts can change the human behavior in real time, the social distancing and mask adoption. This is the explanation why lags of dependent variables must be added in our model to control reverse causality bias. We also tried to add the policy stringency index and the number of tests to take into account confounding and simultaneity. Thus, our empirical work is able to properly make causal identification and not only correlation. In addition, we take into account the presence of some cross-sectional dependence following Chudik et al.^17^, Pesaran^74^ and Chudik and Pesaran^18^ and^19^. Indeed, MG estimator is consistent under the assumption of independent cross-sectional errors. This assumption is likely to be not plausible in our context since the pandemic might have triggered unobserved common shocks that led to cross-sectional error dependence. Thus, we test the weak cross-sectional independence suitable for unbalanced panels^74^ and then relax cross-sectional error independence by following the existing literature and adding contemporaneous and lagged average variables (An alternative way would be to use a GMM estimator in the vein of Anderson and Hsiao, Blundell and Bond, or Arellano and Bond, but they are better suited for small time dimension panels.). Finally, persistence and multicollinearity are other usual issues in panel studies. We have controlled for both by computing autocorrelations LM (Lagrange Multiplier) tests and VIF/Tolerance ratios after each estimated regression. In Appendix, we also consider other robustness tests related to the specification of our econometric model, the choice of an alternative estimator, and several changes in the sample composition.

### The Cross-section model to investigate face mask adoption

We collect data from different sources (SI Appendix) to estimate a very simple cross-section model of the following form at a country level:

2 $$\begin{aligned} \begin{aligned} y_{i} = \alpha _{0} + \alpha _{1} X_{i} + \varepsilon _{i} \end{aligned} \end{aligned}$$

where $y_{i}$ captures proportion of individuals declaring wearing a face mask on 15th of July, 2020 and $X_{i}$ is a set of control variables including population density, CO2 emissions (and squared CO2 emissions), GDP level (in logarithms), government effectiveness index, altruism index, individualism index, tolerance index, risk aversion index, trust in government index, Covid-19 policy stringency index, urban population percentage, education level, diabetic population percentage and overweight population percentage have been considered. We also assume that the dynamics of the epidemics proxied by the count of cases is also a potential driver of masks wearing propensity. We also test as a robustness check the same model with mask wearing data on April, 23, 2020 corresponding to the first available and oldest data from the Maryland survey. Other variables (overweight part of the population, testing, surveillance, travel restrictions and school closures policies) have been introduced (see in SI Appendix).

## The mask adoption is effective on a global scale

We first conducted an econometric exercise for 96 countries for the period April, 23 to July, 15, 2020. To account for lags in epidemic dynamics, we collected data on infected cases and fatalities for a longer period from January, 1, 2020. Our global panel data covers all parts of the globe, but we narrow this down to European countries for robustness checks.

**Figure 1:**
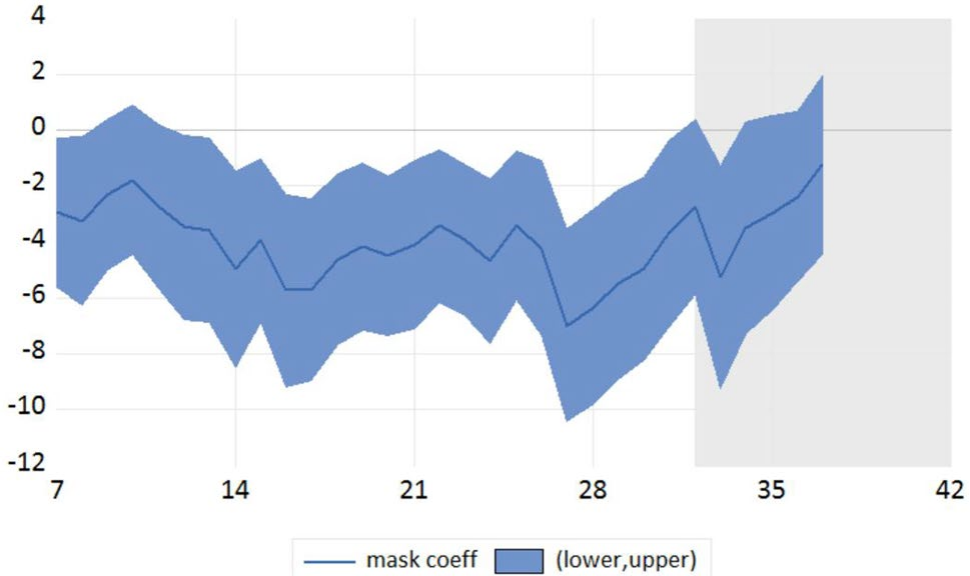
Dynamic effects of face mask use on infected cases case *Note* The X-axis refers to the number of lags/days between the time people declaring wearing a mask and the Covid-19 infected cases number measurement and Y axis refers to the coefficient value with confidence interval in a linear model (not logarithm) with a 10e-08 unit. It is noted that Fig. 1 shows the estimated impact of masks at some particular lag/day and not a sequence of dynamic mask effects like in a Vector AutoRegressive (VAR) or Local Projection (LP) framework.

Our dynamic panel data estimates (Mean Group here) reveal that masks wearing has a clear ($P < 0.01$) statistical negative impact on infected cases with a 7- to 28-day lag but that this negative effect disappears after 42 days (Table 1). The coefficients of the *masks* variable have been standardized (multiplied by 100,000) in Table 1 and Tables 3 , 4, 5 6 to facilitate the reading. The most significant negative effect is obtained after a lag of around 27 days (Fig. 1). For 32 days (the gray area in Fig. 1), the coefficient associated with the mask use variable becomes insignificant. It is noted that Fig. 1 shows the estimated impact of masks at some particular lag/day and not a sequence of dynamic mask effects.

Note that the weak magnitude of the coefficients is explained by the definition - per capita - of the Covid-19 outcomes (cases and fatility rates) and the epidemiological dynamic panel model used (the AutoRegressive part and the deterministic trend(s) capture a high proportion of the variance of the Covid-19 outcome variable). We conducted the same analysis with a log-log econometric model to derive elasticity’s values (Table 2). The coefficients are -0.117 and -0.135 for 7 and 14 lags respectively; in other words, an increase of 100% of the proportion of people declaring wearing a mask (multiply by two) over the studied period lead to a reduction of around 12 and 13.5% of the number of infected cases (per capita) after 7 and 14 days respectively.

We also found a statistical negative impact of mask wearing on fatality rates with a longer lag. Here, we consider 14- and 28-day lagged variables for illustration (Table 3). We also document that the mask effects on deaths for 7 days after are not reported since this effect is not significant. This insignificance is a proof of credibility of our results since the mask has no effect on fatalities at a very short run horizon (Placebo test). The mask use effect on fatalities for a very large horizon (42 days) is also not significant.

As a consequence, the mask wearing effect is more important on infections in the short term (7 to 28 days), whereas the effect is greater on fatality rates in the medium term (14 to 35 days). This is consistent with the epidemiological rationale that mask protection today can reduce the probability of fatalities after approximately one month, which is based on the dynamics of the epidemic, the incubation period, and the length of time before infection, symptoms, and complications are declared. The lag is thus greater for fatalities than for confirmation of infections.

We conducted a variety of robustness checks showing that the negative effect of masks is negative and significant with a small magnitude of the coefficients. Considering the fact that the Covid-19 epidemic did not begin at the same time on all continents, we considered the case of European countries for which data set is available as a homogeneous and robust sample (in SI Appendix, Tables S5–S6 and Tables S10–S12). The robustness of the sample is supported by the fact that European countries report a particularly high number of Facebook panel responses for the mask wearing variable. We also estimate our model for non European Countries (Tables S7 and S8 in SI Appendix) and Asian countries (Table S9 in SI Appendix). The mask adoption is effective and reduces the number of cases and fatalities for credible horizons (7 to 28 days) for both European and non European countries. Surprisingly, this effect is not robust when only Asian countries are considered but this result could be explained by the strong heterogeneity among countries (Gulf countries, Russian countries, South-East Asian countries are blended in the same subsample).

We also accounted for potential omitted bias and collinearity between mask wearing and other mitigation measures, such as testing policies, school closures, and travel bans. We disentangled the effects of mask wearing from those of other mitigation measures (Table 4) and found a robust negative effect of mask use on the Covid-19 outbreak, taking into account the daily number of new tests per thousand of population and the policy stringency index. The mask wearing effect appears to be robust to the introduction of other controls (and potential omitted variables), such as the seasonality and the meteorological conditions by controlling the temperatures (see Carleton et al. (2021) or Damette et al.^21^ about weather and Covid-19) and other mitigation measures, including Covid-19 testing policy and the Covid-19 policy stringency index to control for endogeneity/reverse causality issues (Table 5).

Furthermore, we tested a potential over-controlling problem by dropping some lags and keeping more parsimonious models (Table 6). Indeed, controlling for case rate (t-1) could lead to over-controlling issues. For example, given the uncertainty about the delay/lag between masking use and cases or fatalities, it is possible that case rate (t-1) could be affected by masks (t-7), and very likely that case rate (t-1) would be affected by longer lags associated to the masks variable. Finally, we take into account the presence of some cross-sectional dependence (Table 6) following Chudik et al.^17,74^, and Chudik and Pesaran^18^ and^19^. The Pesaran^74^ test (see the result of the notes in Table 6) indicates the rejection of the null hypothesis of weak cross-sectional dependence. The effect of masks use with a 28 days lag is always significant when lags overcontrolling and cross-sectional dependence are taken into account; however, the results with a 14 days lag are not completely robust. This result is however rationale.

We also considered individual mobility measures - see Weill et al.^88^ and Soucy et al.^82^ for relevant studies about Covid and mobility - from mobile device location pings (here, walking and driving indexes from Apple Trend Reports), see in appendix (Table S12 in SI Appendix). Finally, we check potential reporting bias in our masks variables based on survey respondents by testing a dummy variable as an alternative proxy of mask adoption. Dummy takes 1 when the country introduced a mask requirement policy and zero instead (see Table S24 in SI appendix). It means that our results hold robust when using other mask use proxies (for our benchmark 14 lags model), here a macro policy requirement index instead of individual declarations by respondents of the Facebook survey.

**Table 1:** Masks effects on Covid-19 infected cases: linear model.

	(1)	(2)	(3)	(4)
	Cases rate	Cases rate	Cases rate	Cases rate
Cases rate (t-1)	0.148***	0.135***	0.087***	0.022
	(0.0295)	(0.0285)	(0.0275)	(0.0288)
Cases rate (t-14)	0.118***	0.106***	0.118***	0.142***
	(0.0218)	(0.0226)	(0.0266)	(0.0318)
Masks (t-7)	−0.003**			
	(0.001)			
Masks (t-14)		−0.005***		
		(0.002)		
Masks (t-28)			−0.006***	
			(0.002)	
Masks (t-42)				−0.001
				(0.001)
Time	3.37e-08**	3.94e-08**	7.02e-08***	1.01e-07**
	(1.68e-08)	(1.70e-08)	(2.25e-08)	(4.28e-08)
Constant	−0.0007**	−0.0009**	−0.0015***	−0.0022**
	(0.0004)	(0.0004)	(0.0005)	(0.0009)
Observations	7,359	6,687	5,343	3,999
Number of countries	96	96	96	96

Standard errors are in (.) with *** *p*<0.01, ** *p*<0.05, * *p*<0.1. $t-k$ denotes current time minus *k* days. As an example, ’$Masks (t-7)$’ is the one week lag variable about mask adoption (percentage of the population of a given country). In other words, how a mask adoption today (in time t) is likely to protect a person one week later ($t+7$) considering notably the incubation period. Similarly, ’$Masks (t-14)$’ and ’$Masks (t-28)$’ consider the response after 14 and 28 days respectively. The coefficients for *Masks*’ have been multiplied by 100,000 for standardization purposes. ’$cases rate (t-1)$’ is the reported number of Covid-19 infected people with a one-day lag. It enables to take into account inertia and persistent effects in the dynamics of the epidemics.

**Table 2:** Masks effects on Covid-19 infected cases: log-log model.

	(1)	(2)	(3)
	Cases rate	Cases rate	Cases rate
Cases rate (t-1)	0.272***	0.268***	0.243***
	(0.0240)	(0.0248)	(0.0255)
Cases rate (t-14)	0.0773***	0.0722***	0.0758***
	(0.0187)	(0.0179)	(0.0188)
Masks (t-7)	−0.117*		
	(0.0679)		
Masks (t-14)		−0.135**	
		(0.0579)	
Masks (t-28)			−0.00367
			(0.0550)
Time	0.0079***	0.0084***	0.0088***
	(0.0021)	(0.0022)	(0.0027)
Constant	−180.8***	−192.7***	−200.5***
	(45.60)	(48.20)	(59.56)
Observations	5,985	5,430	4,305
Number of id	91	91	90

Standard errors are in (.) with *** *p*<0.01, ** *p*<0.05, * *p*<0.1.

**Table 3:** Masks effects on Covid-19 fatalities.

	(1)	(2)	(3)
	Fatality rate	Fatality rate	Fatality rate
Fatality rate (t-1)	−0.0023	−0.0338**	−0.0651***
	(0.0201)	(0.0159)	(0.0182)
Fatality rate (t-14)	0.0308*	0.0303*	0.0352
	(0.0170)	(0.0182)	(0.0232)
Cases rate (t-14)	0.0007	4.39e-06	−0.0003
	(0.0006)	(0.0007)	(0.0006)
Masks (t-14)	−0.00006*		
	(0.00004)		
Masks (t-28)		−0.0001***	
		(0.00004)	
Masks (t-42)			−0.00006
			(0.00006)
Time	−5.69e-11	1.09e-10	8.25e-10
	(4.84e-10)	(6.24e-10)	(1.18e-09)
Constant	1.48e-06	−2.28e-06	−1.81e-05
	(1.07e-05)	(1.38e-05)	(2.61e-05)
Observations	6,687	5,343	3,999
Number of countries	96	96	96

Standard errors are in (.) with *** *p*<0.01, ** *p*<0.05, * *p*<0.1. The coefficients about mask effects on fatalities for 7 days is not significant: -7.63e-10 (4.97e-10). The coefficients for *Masks*’ have been multiplied by 100,000 for standardization purposes.

**Table 4:** Masks effects on Covid-19 infected cases and fatalities by controling testing policy.

Variables	(1)	(2)	(3)	(4)
	Cases rate	Cases rate	Fatality rate	Fatality rate
Cases rate (t-1)	0.161***	0.107***		
	(0.0394)	(0.0284)		
Cases rate (t-14)	0.115***	0.117***	0.0008	0.0007
	(0.0269)	(0.0236)	(0.0007)	(0.0006)
Fatality rate (t-1)			0.0007	−0.0152
			(0.0275)	(0.0204)
Fatality rate (t-14)			0.0111	0.0281*
			(0.0183)	(0.0170)
Masks(t-14)	−0.006***	−0.0057***	−0.0001**	−0.00007*
	(0.0023)	(0.0017)	(0.00005)	(0.00004)
New tests number per thousand (t-14)	−1.58e-07		1.26e-07***	
	(6.55e-07)		(4.23e-08)	
Stringency index (t-14)		9.93e-11		−5.99e-10
		(2.21e-08)		(6.23e-10)
Time	4.90e-08**	4.90e-08***	−1.30e-09	−4.11e-10
	(1.97e-08)	(1.86e-08)	(8.96e-10)	(5.45e-10)
Constant	−0.0011**	−0.0011***	2.89e-05	9.40e-06
	(0.0004)	(0.0004)	(1.98e-05)	(1.21e-05)
Observations	4,351	6,596	4,351	6,596
Number of countries	68	95	68	95

Standard errors are in (.) with *** *p*<0.01, ** *p*<0.05, * *p*<0.1. The coefficients for *Masks*’ have been multiplied by 100,000 for standardization purposes.

**Table 5:** Masks effects on Covid-19 outcomes by controlling other policies.

Variables	(1)	(2)	(3)	(4)	(5)
	Cases rate	Cases rate	Cases rate	Fatality rate	Fatality rate
Cases rate (t-1)	0.147***	0.126***	0.078*		
	(0.039)	(0.039)	(0.042)		
Cases rate (t-14)	0.103***	0.124***	0.130***	0.0008	
	(0.027)	(0.031)	(0.035)	(0.0007)	
Cases rate (t-28)					0.0018
					(0.0012)
Fatality rate (t-1)				−0.0172	−0.0589***
				(0.0269)	(0.0202)
Fatality rate (t-14)				0.00610	
				(0.0186)	
Fatality rate (t-28)					0.00657
					(0.0196)
Masks(t-14)		−0.0066***		−0.0002**	
		(0.0021)		(0.0001)	
Temperatures (t-14)		7.840			
		(2.760)			
Stringency index (t-14)		2.560		−6.90e-10	
		(2.320)		(7.70e-10)	
New tests number (t-14)		2.180		1.21e-07***	
		(9.920)		(3.89e-08)	
Masks(t-28)					−0.0001*
					(0.0001)
Temperatures (t-28)			2.160		
			(5.280)		(6.08e-10)
Stringency index (t-28)			3.340		8.26e-10
			(2.830)		(1.08e-09)
New tests number (t-28)			80.660		−1.66e-08
			(176.000)		(6.51e-08)
Time	1.180	7.640***	3.670	−2.02e-09**	9.63e-10
	(2.470)	(2.770)	(3.070)	(1.02e-09)	(1.03e-09)
Constant	−0.001	−0.001***	−0.001	4.55e-05**	−2.08e-05
	(0.001)	(0.001)	(0.001)	(2.25e-05)	(2.26e-05)
Observations	4,519	4,091	3,202	4,343	3,454
Number of countries	67	67	67	67	67

Standard errors are in (.) with ***p<0.01, **p<0.05, *p<0.1. The coefficients for *Masks*’ have been multiplied by 100,000 for standardization purposes.

**Table 6:** Masks effects on Covid-19 fatalities: parcimonious model and cross-sectional dependence.

Variables	(1)	(2)	(3)	(4)
	Deathpop (cross)	Deathpop (cross)	Deathpop	Deathpop
Fatality rate (t-1)	.03553	−0.0625***		
	(.0434)	(.0261)		
Fatality rate (t-14)	.0940***		0.0357*	
	(.0262)		(0.0183)	
Case rate (t-14)			0.0007	
			(0.0006)	
Fatality rate (t-28)		0.0817***		0.0385**
		(0.0305)		(0.0185)
Case rate (t-28)				0.0018**
				(0.0008)
Masks (t-14)	−0.0006		−0.0007	
		(0.0006)	(0.00005)	
Masks (t-28)		−0.0011**		−0.0001***
		(0.0005)		(0.00005)
Time	.0002087	2.74e-08	−1.16e-10	1.36e-09*
	(.0002087)	(2.71e-08)	(5.38e-10)	(7.12e-10)
Constant	.00006	.00004	2.66e-06	−2.97e-05*
	(.0001)	(.0001)	(1.19e-05)	(1.57e-05)
Observations	6,495	4,863	6,687	5,343
Number of id	96	96	96	96

Standard errors are in (.) with *** *p*<0.01, ** *p*<0.05, * *p*<0.1. Pesaran^74^ cross-section dependence test: CD = 4.313 *p* value = 0.000. 2 and 5 lags of supra average variables have been included respectively. In the two first columns, ’(Cross)’ indicates that cross-sectional dependence has been taken into account by adding supra averages in MG regressions. The coefficients for *Masks*’ have been multiplied by 100,000 for standardization purposes.

## The main drivers of mask wearing

As demonstrated in the previous section, face mask use is negatively correlated with infections and fatality rates. The next question is why individuals in some countries are more likely to wear masks wearing than those in other countries. Since the main characteristics of the countries are fixed over time, it was only possible to conduct descriptive statistics investigations and cross-sectional regressions. Based on information from scatter plots, a correlation coefficients table, partial regressions (see Tables S22 and following ones in SI Appendix) and multivariate cross-section regressions, we can report some interesting insights.

**Figure 2:**
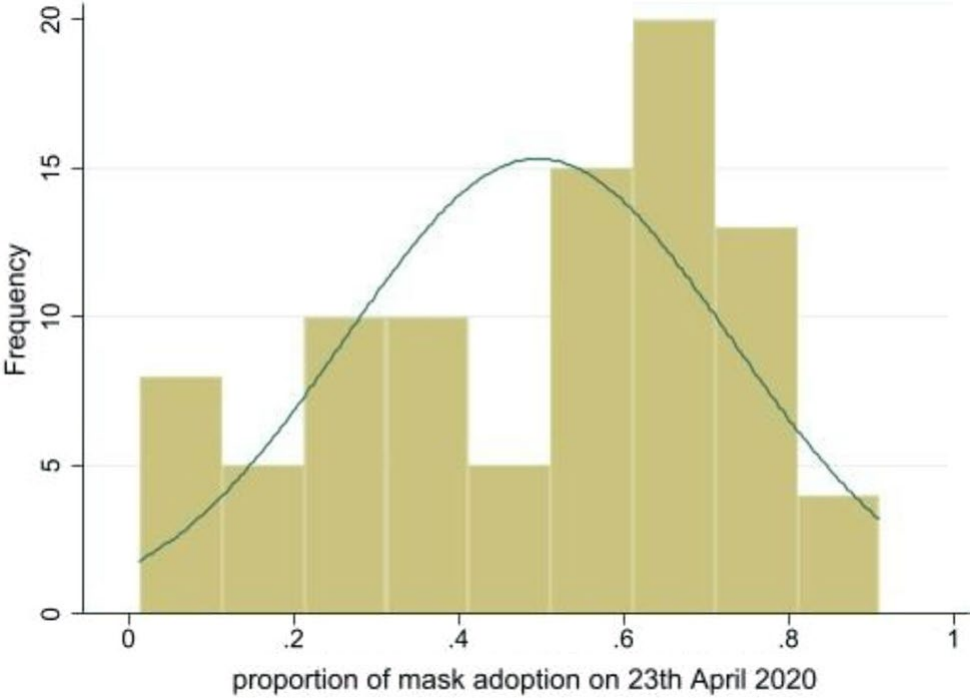
Distribution of masks wearing across countries on 23th April, 2020 *Note* The X-axis refers to the proportion (between 0 and 1) of individuals declaring wearing a mask on the beginning of our sample (23th April, 2020) and Y axis refers to the histogram (frequency). The normal distribution curve is presented as a benchmark.

**Figure 3:**
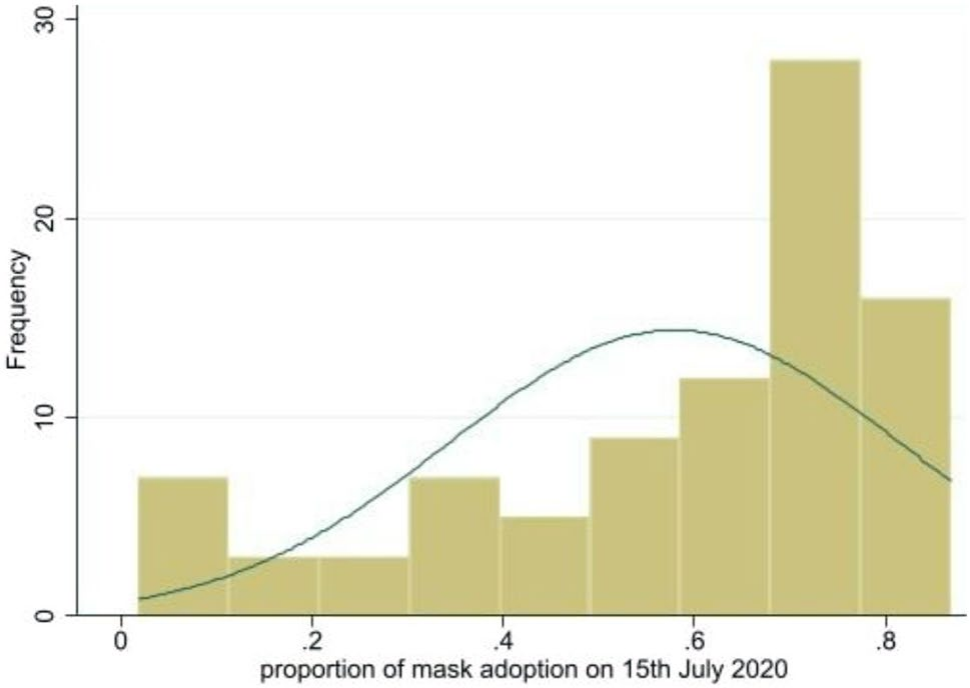
Distribution of masks wearing across countries on 15th July, 2020 *Note* The X-axis refers to the proportion (between 0 and 1) of individuals declaring wearing a mask on the last date of our sample (15th July, 2020) and Y axis refers to the histogram (frequency). The normal distribution curve is presented as a benchmark.

First, we found that individuals appear to have implicitly understood the crucial role of mask wearing by themselves since we note a convergence to higher levels of mask wearing across countries over time. This is indicated in Figs. 2 and 3 by the fact that the distribution of the mask wearing variable moves to the right as we move from the initial period (April, 23 or 24) to the final period (July, 15). In other words, more and more individuals declared on Facebook that they had adopted mask wearing to combat the Covid-19 virus. The increasing dynamics of the pandemics, the communication (both public and private) on the role of masks in reducing transmission, and the changing of habits are potential explanations for this result.

**Figure 4:**
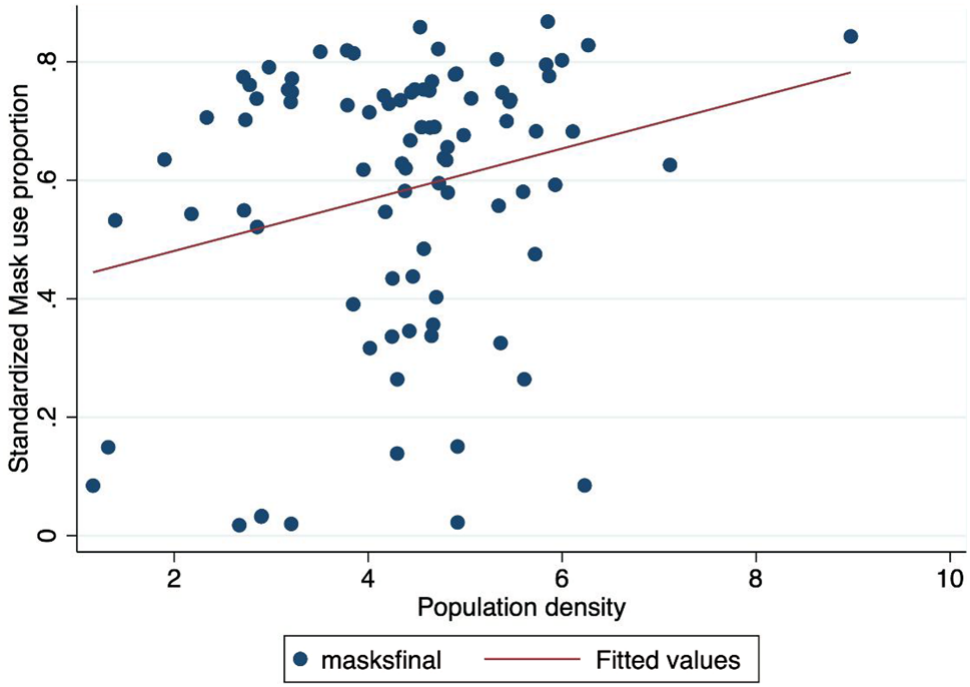
Mask adoption versus population density *Note* The X-axis refers to the mask adoption proportion: a 0.8 value means that 80 respondents over 100 declared to use a face mask. The Y-axis denotes the population density that is the number of people per squared km. ’maskfinal’ denotes the proportion of people using a face mask in the final observation of our sample (15th July, 2020). The red line refers to a simple linear fit. A positive correlation indicates that a higher mask use proportion is associated with a higher population density.

We then estimated the determinants of mask adoption on a global scale by screening a set of potential predictors. The cross-sectional results are presented in Tables 7 and  8. We found that population density was positively associated (Fig. 4, Tables 7–8) with the percentage of individuals wearing a face mask daily. This effect is generally robust ($P < 0.01$ to $P < 0.1$). Mask adoption was highest in countries with a high population density and lowest in countries with a low population density. The literature shows that mask wearing is usually viewed as a complement to social distancing measures; therefore, using masks as a protective measure is more important in densely populated environments (e.g., urban areas, public transport, supermarkets, town centers) than in less densely populated regions (e.g., forests, rural areas). In state capitals and large cities, mask wearing is generally mandatory, especially on public transport. However, when we tested the urban population percentage variable directly, we found the results were less clear cut and that the associated coefficient was never significant. Population density in general seems to be more important than the urban population percentage.

**Figure 5:**
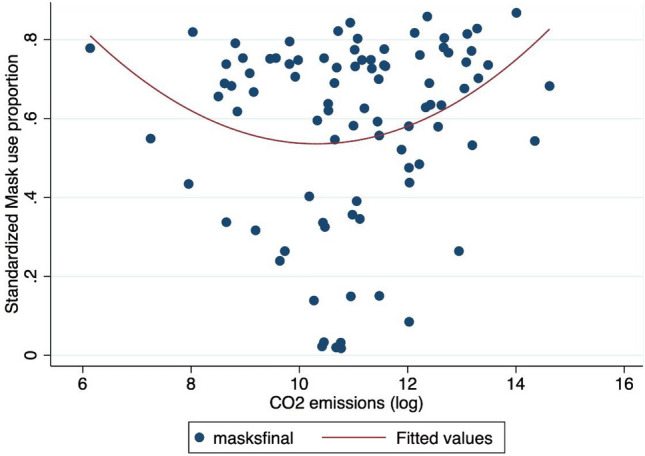
Mask adoption versus CO2 emissions *Note* The X-axis refers to the mask adoption proportion: a 0.8 value means that 80 respondents over 100 declared to use a face mask. The Y-axis denotes the CO2 emissions (in log). ’maskfinal’ denotes the proportion of people using a face mask in the final observation of our sample (15th July, 2020). The red line refers to a quadratic fit.

Another main driver of mask wearing on a global scale is the level of pollution. Indeed, pollution levels proxied by CO2 emissions were positively associated ($P < 0.01$ in most cases) with the proportion of mask users only for high levels of CO2 emissions, but this effect was reversed for low levels of emissions (Fig. 5). It is probable that inhabitants of countries with high levels of pollution are more likely to wear masks to fight Covid-19 because they are in the habit of using them as a protective measure against harmful particles. Indeed, in some countries, face masks are used to reduce the negative effects of pollution in normal ‘non-Covid-19’ times. Thus, the marginal cost of adopting mask wearing behavior in those countries is low or null. No change of habits is required for individuals from these countries as they just continue to wear masks as they had done prior to the pandemic. Recent studies have also shown that mask wearing is especially necessary in highly polluted environments as Covid-19 fatality rates are exacerbated by high levels of pollution^20^.

**Figure 6:**
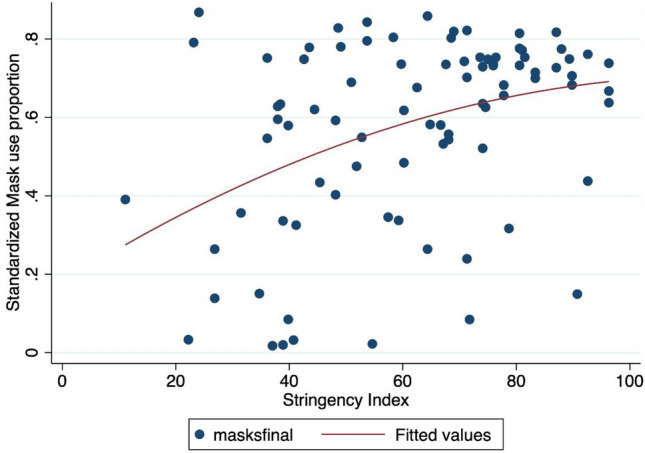
Mask adoption versus policy stringency index *Note* The X-axis refers to the mask adoption proportion: a 0.8 value means that 80 respondents over 100 declared to use a face mask. The Y-axis denotes the Stringency index. ’maskfinal’ denotes the proportion of people using a face mask in the final observation of our sample (15th July, 2020). The red line refers to a quadratic fit.

The most important driver of the level of mask wearing in a given population was the Covid-19 stringency index. This index, compiled by^35^, measures the stringency of government responses to the Covid-19 pandemic across the world based on nine indicators: a higher score indicates a stricter government response, here on July, 15, 2020. This index was positively correlated with mask wearing, and this effect was particularly robust ($P < 0.001$). In other words, when government responses are stricter, mask use is significantly higher (Fig. 6). If the results we report in the previous section are correct - that is, if increasing mask use reduces the negative consequences of the Covid-19 virus - the more stringent countries may obtain better results in fighting the pandemic. We also tested the lagged stringency index (one month lagged variable) and the growth rate of the index over the month previous to the date of variables counting (July, 15, 2020).

In addition, we tested whether the proportion of vulnerable individuals in a population affected the likelihood of individuals to adopt mask wearing to protect themselves and others. Linear partial regressions (see Appendix, Tables S14 and S16 in SI Appendix) show that a high proportion of diabetic individuals in a population is positively associated with mask wearing. However, this effect appears not to be robust in a multivariable framework. Similarly, results are inconclusive regarding the proportion of overweight individuals variable though the coefficient attached to this variable is significant in canonical regressions in a nonlinear way (see in SI Appendix).

Government effectiveness and GDP appear to be negatively correlated ($P < 0.01$ in most cases) with the proportion of people wearing masks. This is surprising since we expected that rich countries would be more likely to make masks free of charge or subsidize part of the cost and that individuals in these countries would have higher incomes and, therefore, less economic constraints to prevent them from buying the required quantity of face masks to protect them in public and private areas. Regarding the government effectiveness variable, countries with a high level of government effectiveness are generally characterized by better policy formulation and implementation and higher credibility of government commitment, which is likely to increase the probability of mask wearing and higher compliance with government policy. Indeed, it has been found that strong public guidance is necessary to promote and enforce mask wearing^26^ and also^9^. It should be noted countries with high a GDP and those with a high government effectiveness are likely to be similar (the correlation is around 0.81 between these two variables), which explains why both coefficients are moving in the same direction. To avoid multicollinearity issues, we alternatively added government effectiveness or GDP in the tested models.

We expected that countries with a high proportion of elderly individuals are expected would have a high level of mask wearing, based on previous findings from^85^. However, the sign of association between masks and the proportion of elderly individuals was negative in partial regressions (see SI Appendix) and not significantly robust in multivariable regressions. There are several possible explanations for this: countries with a high proportion of individuals aged over 65 are generally industrialized countries in Europe where, unlike in Asian countries with younger populations , mask wearing is not habitual. It is possible that older individuals, whose flexibility and capacity for adaptability are generally lower, are less likely to wear masks. Countries with a high proportion of elderly individuals are also likely to be more conservative and, thus, their inhabitants may take longer to subscribe to new habits and rules. In a letter, some authors underlined that socially responsible behaviors may not be intuitive and that behavior change takes time^66^. This is probably truer for countries with older populations . Our multivariate regressions results do not indicate any significant negative relationships, which suggests that demographics structure is probably not a key factor affecting mask wearing disparities across countries.

It is also crucial to consider how the current dynamics of the pandemic might influence the habits of individuals regarding mask wearing and the adoption of other social measures to reduce the transmission rate. Public information on the dynamics and intensity of the Covid-19 epidemic is probably an important driver of mask wearing, and this variable indirectly captures this effect. When the number of cases is increasing, individuals are more informed and aware of the epidemic’s negative consequences and are, therefore, probably more inclined to take control measures such as mask wearing and social distancing more seriously. The higher the rate of infected cases, the higher the probability that individuals will personally know infected individuals (e.g., neighbors, family members, work colleagues). This may make individuals perceive the virus as more frightening and lead to increasing levels of social responsibility, making individuals more likely to wear a mask and obey rules to reduce the transmission rate. We found that the link between the infection count (we used the official count on July, 15, 2020 but also tested a lagged variable) and mask wearing was positive but not always significant at high confidence levels (Table 8).

**Figure 7:**
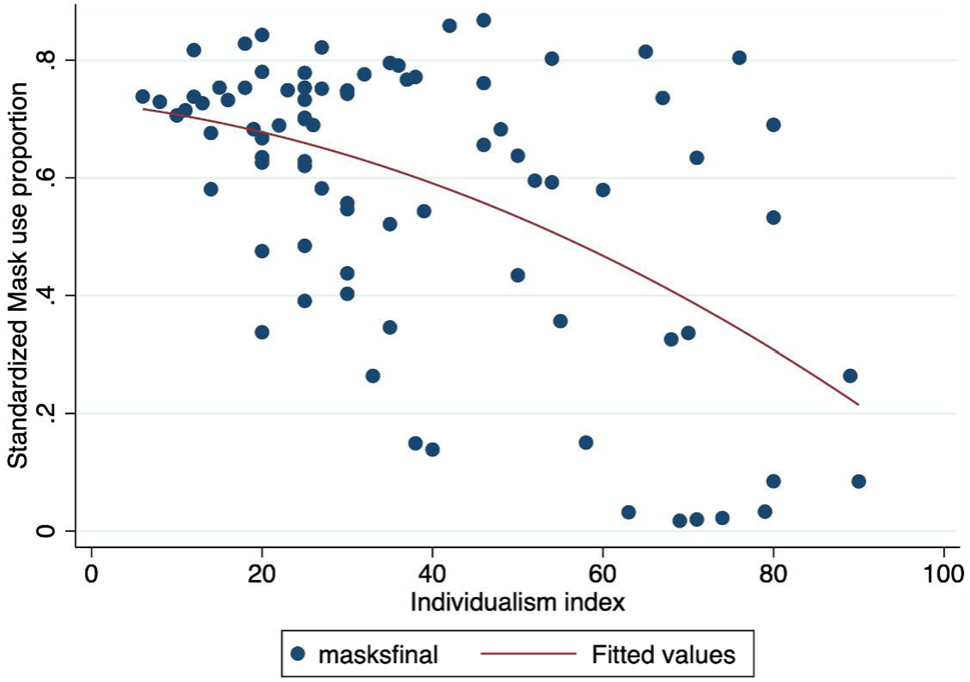
Mask adoption versus individualism index *Note* The X-axis refers to the mask adoption proportion: a 0.8 value means that 80 respondents over 100 declared to use a face mask. The Y-axis denotes the Individualism index. ’maskfinal’ denotes the proportion of people using a face mask in the final observation of our sample (15th July, 2020). The red line refers to a quadratic fit.

Finally, we attempted to evaluate whether individuals in countries with higher levels of altruism, solidarity, and tolerance were more likely to use masks. A scatter plot showed a very modest positive association between mask wearing and the recent (2018) altruism index of Falk and co-authors (Falk et al., 2020). However, we found no clear-cut correlation, especially in our multivariable analysis (Table 6). We also tested the effect of the tolerance index from the World Value Survey database. Tolerance level was significantly associated with mask use for countries with low to intermediate tolerance values, but the association was negative for countries with high tolerance levels. This effect may be due to the fact that Scandinavian countries, which are characterized by high levels of tolerance, have implemented liberal policies on mask wearing . We also test if the trust in politicians and government can affect the mask wearing proportion in line with^5^ but do not find a clear significant relationship: in other words, we do not find that a high proportion of people that do not trust in government ’at all’ is associated to a weak proportion of mask wearing in the population but due to the limited number of observations, the inference work should be interpreted with cautious. More generally, note that the number of available observations for regressions including altruism and tolerance variables is limited (around 50 observations). Finally, we test the effect of individualism with a more larger data set (77 observations in column (5) from Table 8) in line with Ozkan et al.^70^ who find a positive relationship between individualism (defined as preference of loosely-knit social framework from Hofstede Insights) and Covid-19 severity. We find evidence that individualism is negatively correlated to mask use (Table 6) as also shown by the Fig. 7. This result is interesting, novel and imply that people in countries with high level of individualism are less likely to wear mask and maybe to respect social distancing rules.

**Figure 8:**
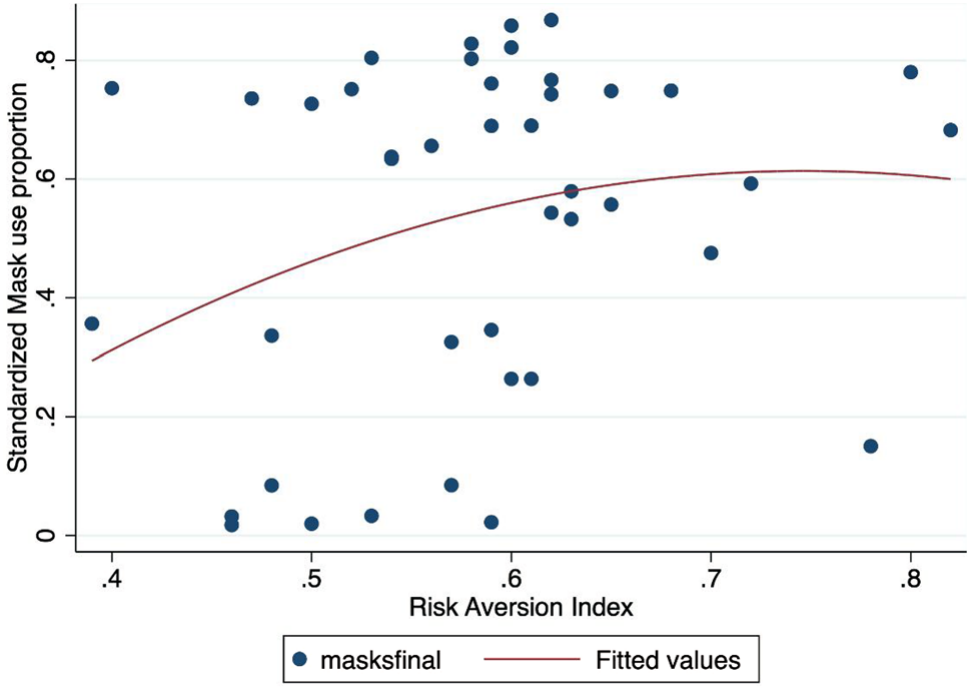
Mask adoption versus risk aversion index *Note* The X-axis refers to the mask adoption proportion: a 0.8 value means that 80 respondents over 100 declared to use a face mask. The Y-axis denotes the risk aversion index. ’maskfinal’ denotes the proportion of people using a face mask in the final observation of our sample (15th July, 2020). The red line refers to a quadratic fit.

Although we expected that education levels might affect the learning of useful hygiene rules and suitable behaviors to confront contagious diseases, education level (schooling variable from the World Bank or Pisa score) was found to have a non-significant negative effect on mask wearing (see SI Appendix, Table S18 in SI Appendix). Same result is obtained for the risk aversion (ambiguity index) computed by Ruieger (2015): the positive correlation between mask adoption and high risk aversion (Fig. 8) seems clear-cut and confirm that risk averse people are more prone to use face masks. Nevertheless, the positive coefficient turns to be insignificant when controls like population over 65 are included in the regression. However, note that the number of available observations is very very small (around 40-45 according to the specifications), so we need to take the econometric results with cautious.

In sum, our results complement those of previous studies, extending them from the level of individual surveys to the country scale. While previous studies investigated factors such as age, gender, or threat perception, our study explores the role of country-level macroeconomic and socio-economic determinants in explaining the substantial heterogeneity across countries regarding the wearing of face masks.

**Table 7:** Cross-crountry determinants of mask covering (1).

Variables	(1)	(2)	(3)	(4)	(5)
	Masks	Masks	Masks	Masks	Masks
Population density	0.035*	0.039*	0.052**	0.033*	0.045**
	(0.019)	(0.019)	(0.020)	(0.019)	(0.018)
co2 level	−0.328***	−0.300***	−0.344***	−0.319***	−0.343***
	(0.106)	(0.102)	(0.110)	(0.101)	(0.106)
co2$^2$ level	0.015***	0.014***	0.016***	0.015***	0.016***
	(0.004)	(0.004)	(0.005)	(0.004)	(0.005)
gdp		−0.046*			
		(0.027)			
Government effectiveness			−0.083***		
			(0.029)		
Urban population				−0.001	
				(0.001)	
Stringency index					0.005***
					(0.001)
Constant	2.098***	2.350***	2.065***	2.092***	1.829***
	(0.529)	(0.634)	(0.549)	(0.529)	(0.514)
Observations	88	87	85	88	85
R-squared	0.105	0.142	0.227	0.108	0.310

Standard errors are in (.) with *** $\hbox {p}<0.01$, ** $\hbox {p}<0.05$, * $\hbox {p}<0.1$.

**Table 8:** Cross-crountry determinants of mask covering (2).

Variables	(1)	(2)	(3)	(4)	(5)
	Masks	Masks	Masks	Masks	Masks
Population density	0.048**	0.056***	0.041	0.033	0.041**
	(0.019)	(0.020)	(0.026)	(0.034)	(0.017)
CO2 level	−0.335***	−0.383***	−0.287**	−0.311	−0.392***
	(0.113)	(0.120)	(0.131)	(0.302)	(0.112)
CO2$^2$ level	0.016***	0.018***	0.014**	0.015	0.019***
	(0.005)	(0.005)	(0.006)	(0.013)	(0.005)
Stringency index	0.004***	0.004***	0.006***		0.004***
	(0.001)	(0.001)	(0.001)		(0.001)
Diabetic proportion	0.001	0.002			−0.002
	(0.006)	(0.006)			(0.005)
Proportion of 65 elderly	0.001	0.005			0.007*
	(0.005)	(0.005)			(0.004)
Cumulated cases	0.049	0.057	0.024	0.127*	0.034
	(0.043)	(0.034)	(0.085)	(0.079)	(0.024)
gdp	−0.023				
	(0.034)				
Gov effectiveness		−0.070*			
		(0.035)			
Altruism			0.028		
			(0.064)		
Tolerance				0.048**	
				(0.020)	
Tolerance$^2$				−0.001***	
				(0.001)	
Individualism					−0.006***
					(0.001)
Constant	1.992***	1.957***	1.429**	0.574	2.237***
	(0.705)	(0.599)	(0.643)	(1.887)	(0.637)
Observations	82	80	54	49	77
R-squared	0.333	0.362	0.352	0.485	0.537

Standard errors are in (.) with ***p<0.01, **p<0.05, *p<0.1.

## Discussion

Understanding the links between face mask use and Covid-19 is crucial since the effectiveness of masks has been widely debated and contested among the general public. Our panel econometric exercise demonstrates that the wearing of face masks is negatively associated with infections and fatalities at the country level. Therefore, mask wearing has an important role to play in controlling the spread of Covid-19. Given the effectiveness of mask wearing in significantly curbing the transmission of the airborne Covid-19 virus and, thus, reducing the number of infections and fatalities, it would be helpful for governments to focus more on promoting the use of masks than on invoking the precautionary principle. We document a process of convergence over time in favor of mask adoption by showing the increasing percentages of people wearing masks across countries. This is good news as it suggests that individuals have implicitly understood the substantial impact of mask use on fighting the Covid-19 pandemic. However, our data shows that the mean proportion of inhabitants wearing masks was only 56% on July, 15, with a large standard deviation. Even in countries with high levels of mask use, the number of individuals that avoid mask wearing is probably still too large.

Appropriate public hygiene and control policies would consist of mandatory mask wearing. Given the clear effect of mask wearing on infections and fatalities and the fact that a mandatory mask policy involves little economic disruption (Chernozhukhov et al., 2020) and is economically attractive, we also recommend extending mandatory face mask use even for children (over 6 years of age). Attitudes regarding altruism, tolerance, and solidarity do not appear to be sufficient to achieve the necessary levels of mask wearing. In contrast, individualism is negatively correlated to mask use. In addition, strict government responses significantly increase levels of mask use. In this respect, our results are in line with the findings of^9^, which show that a voluntary policy leads to insufficient compliance. In addition, since the absence of economic constraints that might prevent the purchase of masks in high-income countries seems not to be a key driver, the main reasons for not wearing masks appear to be subjective, cultural, or the result of a lack of legal sanctions. Legally requiring people to wear face masks, thus, appears to be an effective instrument^77^. Therefore, some countries could introduce stronger penalties for not wearing masks. Indeed, the only effective way of enhancing mask adoption and saving more lives appears to be to implement more stringent policies. Our policy implication is also drawing how to nudge people with the different norms due to the heterogeneity in the root of culture. For example, the East Asian countries could consider the calling action for the whole community, as known as collectivism. That’s why Vietnam is doing the good job at the early stage to call for everyone to fight against the pandemic as the national campgain^40,41^.

*Limitation* Since this is the first study that attempts to explore the effects of COVID-19 status by using the mask in the initial stage, the study has not taken into account the medical treatment or pharmaceutical intervention. Future studies could focus on the persistence of using the mask to stop the spread of the virus. Concomitantly, it would be essential to disentangle the separation between using a mask and other protective methods (such as vaccination or using medicines). More importantly, the country’s strategies are also important in shaping human behaviors in wearing a mask. For example, Vietnam has changed its policy from zero COVID to ’living with COVID’^43^, which might significantly change the behavior of wearing a mask. Therefore, it would be a potential avenue indeed.

## Supplementary Information

Supplementary Information.

## Data Availability

The datasets generated and/or analysed during the current study are available from the corresponding author on reasonable request and dat as well as main replication codes are available at https://dataverse.harvard.edu/dataverse/MaskCovid19. A detailed SI Appendix is associated to the main manuscript with detailed informations about data and materials. The survey data come from the study performed by the University of Maryland and jointly conducted with the Facebook platform (see Fan et al.^29^, Barkay et al.^6^ and Kreuter et al.^50^) and are publicly available (see details in SI Appendix).
