# Supplementary material for: Face mask is an efficient tool to fight the Covid-19 pandemic and some factors increase the probability of its adoption
Source: Sci Rep. 2023 Jun 6;13:9218. doi: 10.1038/s41598-023-34776-7 (PMC10243888; doi:10.1038/s41598-023-34776-7)
Supplement: Supplementary file 1 — Supplementary Information. [file 41598_2023_34776_MOESM1_ESM.pdf]

SI Appendix: Face mask is an efficient tool to fight the Covid-19 pandemics and some factors increase the probability of its adoption.

March 11, 2023

1. Variable definition and data sources

For each variable, we indicate the acronym, the definition and the sources respectively. This section consists of two main parts: panel data with two indicators (country - i and daily - t) and cross-sectional data with one dimension (country-level).

1.1. Panel data for each country at daily basis

- *Casepop* and *Deathpop*: Covid-19 cases and deaths on a daily basis have been collected from Johns Hopkins University on July 15, 2020. We compute infected cases and fatality rates by using the cumulative values divided by the total population of country i to obtain a *percapita* ratio at a daily frequency. Our panel data are available from January, 1, 2020 to July, 15, 2020. The *casepop* variable is computed as the following ratio: number of infected cases on day d over total population of the country. The *deathpop* variable is computed as the fatality rate: number of cumulative fatalities on day d over total population of the country i. To take into account the dynamics of the epidemics, the incubation period (time between t and $t-k$) and other delays of action of the virus, we also use lagged $casepop(t-k)$ and $deathpop(t-k)$ variables.

- *New_tests*: This variable refers to the number of new Covid-19 tests at a daily frequency per thousand variable comes from Hasell et al. (2020).

- *Masks*: The data come from the study performed by the University of Maryland and jointly conducted with the Facebook platform (see Fan et al. (2020), Barkay et al. (2020) and Kreuter et al. (2020)). It consists in aggregate weighted estimates masks wearing variable that is computed as the number of individuals reporting a mask use on the Facebook platform. We use a standardized value as a benchmark variable by dividing the mean value of wearing mask by its standard deviation. We get success to collect data for 96 countries although the number of observations varies across countries. As explained by the authors: "We are inviting Facebook app users in more than 200 countries or territories globally to take a survey collected by faculty at the University of Maryland (UMD) Joint Program in Survey Methodology (JPSM). As part of this initiative, we are applying best practices

from survey statistics to design and execute two components: (1) sampling design and (2) survey weights, which make the sample more representative of the general population". "We provide weights for two sets of sample respondents separately for both the CMU US and UMD global surveys. First, we provide weights for respondents who answered the questions needed to calculate the aggregate estimates of COVID-like Illness (CLI) reported in the CMU and UMD APIs. Second, we provide weights for a larger set of respondents who answered a minimum of two questions in the surveys." We extracted data from Maryland University link at https://covidmap.umd.edu/api.html. The surveys ask respondents how many people in their household are experiencing COVID-like symptoms, among other questions. These surveys are voluntary, and individual survey responses are held by University of Maryland and are shareable with other health researchers under a data use agreement. No individual survey responses are shared back to Facebook. Using this survey response data, we estimate the percentage of people in a given geographic region that use face mask cover. We use the smoothed weighted (two sets of sample respondents separately are used (CMU US and UMD global surveys)) percentage of survey respondents across an one week window that have reported use mask cover (see https://covidmap.umd.edu/document/css_methods_brief.pdf). More precisely, we used the following $percent_mc$ variable: weighted percentage of survey respondents that have reported use mask cover. Using the survey data, the authors of the survey estimate the percentage of people in a given country or region, on a given day that use mask cover. The $smoothed_mc$ consisting in seven-day rolling average of $percent_mc$ values as well as $smoothed_mc_se$ that is the standard error of $smoothed_mc$. has also been considered. We finally computed a standardized index: $smoothed_mc/smoothed_mc_se$

- *Stringency*: The Stringency Index summarizes the severity of the lockdown measures. The policy response tracker data has been developed by Oxford's public policy school and the Blavatnik School of Government. They publish it as the Coronavirus Government Response Tracker (OxCGRT). The OxCGRT researchers calculate a summary measure of nine of the response metrics called the Government Stringency Index: school closures; workplace closures; cancellation of public events; restrictions on public gatherings; closures of public transport; stay-at-home requirements; public information campaigns; restrictions on internal movements; and international travel controls. The specific policy and response categories are coded as follows and then each indicator is re-scaled to get a general score between 0 and 100 (100 representing the highest degree of strictness). The detailed components of *stringency* can be found as:

  1. School closures:

     - 0 - No measures.

- ☐ 1 - Recommend closing.
- ☐ 2 - Require closing (only some levels or categories, eg just high school, or just public schools).
- ☐ 3 - Require closing all levels.
- ☐ No data - blank.

2. Workplace closures:
   - ☐ 0 - No measures.
   - ☐ 1 - Recommend closing (or work from home).
   - ☐ 2 - Require closing (or work from home) for some sectors or categories of workers.
   - ☐ 3 - Require closing (or work from home) all but essential workplaces (eg grocery stores, doctors).
   - ☐ No data - blank.
3. Cancel public events:
   - ☐ 0 - No measures.
   - ☐ 1 - Recommend cancelling.
   - ☐ 2 - Require cancelling.
   - ☐ No data - blank.
4. Close public transport:
   - ☐ 0 - No measures.
   - ☐ 1 - Recommend closing (or significantly reduce volume/route/means of transport available).
   - ☐ 2 - Require closing (or prohibit most citizens from using it).
5. Public information campaigns:
   - ☐ 0 - No COVID-19 public information campaign.
   - ☐ 1 - Public officials urging caution about COVID-19.
   - ☐ 2 - Coordinated public information. campaign (e.g. across traditional and social media)
   - ☐ No data - blank.
6. Stay at home:
   - ☐ 0 - No measures.
   - ☐ 1 - Recommend not leaving house.
   - ☐ 2 - Require not leaving house with exceptions for daily exercise, grocery shopping, and 'essential' trips.

☐ 3 - Require not leaving house with minimal exceptions (e.g. allowed to leave only once every few days, or only one person can leave at a time, etc.).

☐ No data - blank.

7. Restrictions on internal movement:

   ☐ 0 - No measures.

   ☐ 1 - Recommend movement restriction.

   ☐ 2 - Restrict movement.

8. International travel controls:

   ☐ 0 - No measures.

   ☐ 1 - Screening.

   ☐ 2 - Quarantine arrivals from high-risk regions.

   ☐ 3 - Ban on high-risk regions.

   ☐ 4 - Total border closure.

   ☐ No data - blank.

9. Testing policy:

   ☐ 0 – No testing policy.

   ☐ 1 – Only those who both (a) have symptoms AND (b) meet specific criteria (eg key workers, admitted to hospital, came into contact with a known case, returned from overseas).

   ☐ 2 – testing of anyone showing COVID-19 symptoms.

   ☐ 3 – open public testing (eg 'drive through' testing available to asymptomatic people).

   ☐ No data - blank.

10. Contract tracing:

    ☐ 0 - No contact tracing.

    ☐ 1 - Limited contact tracing - not done for all cases.

    ☐ 2 - Comprehensive contact tracing - done for all cases.

    ☐ No data - blank.

11. Face coverings

    ☐ 0 - No policy.

    ☐ 1 - Recommended.

    ☐ 2 - Required in some specified. shared/public spaces outside the home with other people present, or some situations when social distancing not possible.

☐ 3 - Required in all shared/public spaces outside the home with other people present or all situations when social distancing not possible.

☐ 4 - Required outside the home at all times regardless of location or presence of other. people

- *Temperatures*: Meteorological conditions consist in mean daily temperatures (in Celcius degrees) from daily have been aggregated by Aviskar Bhoopchand, Andrei Paleyes, Kevin Donkers, Nenad Tomasev, Ulrich Paquet (2020), DELVE Global COVID-19 Dataset and are available at http://rs-delve.github.io/data/global-dataset.html. For each country, all climatic observations have been weighted by the population. In addition, the temperature variable has been standardized (divided by standard deviation) for comparisons purposes and for coefficients scale homogeneity.

- *Mobility*: We use Apple mobility trend reports data at https://covid19.apple.com/mobility. The database consists in two indicators: *Mobility_travel_driving* and *Walking*. Both indicators are indexes (January, 13, 2020 as the 100 basis) and are computed as the % change since the January, 13, 2020 basis. Apple's mobility trend reports show how human mobility has changed in countries and cities worldwide since January 2020 and are based on location data of Apple's "maps" services. It is designed to help mitigate the spread of COVID-19, provide governments, research institutions, health authorities, and the general public with insights on the effects on human mobility of national and regional lockdown policies. The data covers 63 countries. All data are shared on an aggregated level and Apple does not keep a history of users' mobility behaviour. Data from Google reports have also been considered as an alternative. We proceed to some tests and the dynamics from both databases are very similar. Since Apple data are available on a more longer period (January, 13, 2020 versus February, 15, 2020), we have chosen Apple data in our paper. We conducted a set of additional regressions and robustness checks using our panel data of 96 countries to test the effectiveness of mask use and then concerning the cross-sectional work about the mask use determinants.

1.2. Cross-sectional data for country-level

- *Age*65: Population aged over 65 as a percentage of the total population from the World Bank Database. Population is based on the de facto definition of population, which counts all residents regardless of legal status or citizenship.

- *Density*: Population density has been collected from the World Bank Database and is computed as the population divided by land area in square kilometers. Population is based on the de facto definition of population, which counts all residents regardless of legal status or citizenship except for refugees not permanently settled in the country of asylum, who are generally considered part of the population of

their country of origin. Land area is a country's total area, excluding area under inland water bodies, national claims to continental shelf, and exclusive economic zones. In most cases the definition of inland water bodies includes major rivers and lakes.

- *CO2*: Carbon dioxide (CO2) emissions from the World Bank Database are those stemming from the burning of fossil fuels and the manufacture of cement. They include carbon dioxide produced during consumption of solid, liquid, and gas fuels and gas flaring. The natural logarithm of the CO2 emissions has been considered in our econometric regressions.

- *Diabetic*: Diabete measures Diabetes prevalence refers to the percentage of people ages 20-79 who have type 1 or type 2 diabetes complied by the World Bank Database (International Diabetes Federation, Diabetes Atlas).

- *Overweight*: Overweight measures the percentage of the adult population which is overweighted. Data have been collected from Porcher (2021).

  GDP: GDP measures GDP per capita in purchasing power parity for 2017 from the World Bank Database. It controls for income effect: masks are not costless and we expect that income could be positively related to face mask adoption.

- *School*: School enrollment data from the World Bank Database are used to proxy education impact on mask use. Primary school enrollment in % is the ratio of total enrollment, regardless of age, to the population of the age group that officially corresponds to the level of education shown. Primary education provides children with basic reading, writing, and mathematics skills along with an elementary understanding of such subjects as history, geography, natural science, social science, art, and music. Last available year is 2017.

- *PISA*: The Reading performance (PISA) Boys / Girls, mean score in 2018, has been considered in our study. Data have been collected form PISA: Programme for International Student Assessment Data at https://www.oecd.org/pisa/data/.

- *Altruism*: Altruism index has been collected from Falk et al.(2018) and was measured through a combination of one qualitative and one quantitative item, both of which are related to donations. The qualitative question asked respondents how willing they would be to give to good causes without expecting anything in return on an 11-point scale. The quantitative scenario depicted a situation in which the respondent unexpectedly received 1,000 euros and asked them to state how much of this amount they would donate (Table I).

- *Tolerance*: We used the tolerance intentions index from the World Value Survey database. More precisely, we selected answers to the question 12 untitled 'Tolerance and respect for other people' from the survey 2017-2020 untitled 'Here is a list of qualities that children can be encouraged to learn at home. Which, if any, do you consider to be especially important? Please choose up to five'. Our index has been computed by reporting the percentage (over the total of responses) of 'important' responses.

- *Government_confidence*: We also tested the possibility that trust in politicians and government can impact the compliance in line with Bargain and Aminojov (2020). To do that, we used data concerning the question 71 untitled 'Confidence: The government' from the survey 2017-2020 (World Value Survey database). We computed the percentage score concerning the "not at all" answer. In our opinion, it is a mean to capture the proportion of people in a given country that do not trust at all in the government and public guidance and thus could reject public guidance in favor of mask adoption and/or requirements.

- *Risk_aversion*: We collected the risk aversion Degree of Ambiguity Aversion at the Country Level from Rieger et al. (2015). We assume that heterogeneous risk preferences could diverge the behaviors of using masks as well as COVID-19 severity.

2. Effectiveness of mask use: additional regressions and robustness checks

In addition to the robustness tests presented in tables 5-7 in the main manuscript, we also conducted other robustness checks concerning endogeneity and potential omitted issues and by controlling sample biases.

Taking into account additional control policies variables

The existence of a significant correlation between the number of mas wearing people and the number of infected cases and fatalities respectively is a controversial special issue. Some critiques have been addressed to the Zhang et al. (2020) work, especially the fact that the authors do not consider other non pharmaceutical mitigation policies in their statistical analysis as well as the length of the studied sample, notably in the New-York case.

Here, we consider additional control variables and especially include variables about other non pharmaceutical mitigation policies. Considering our time varying panel day by day database, we should incorporate some time varying variables to proxy other policy measures. We first consider the number of new tests per 100k inhabitants to investigate if the mask wearing effect is not driven by the existence of a simultaneous testing policy. We find that the effect of mask wearing is robust to the presence of a 'tests' variable for 14 days in the core of the paper (Table 4) but we extend this test to more lags for the infected cases (Table 9)

and for fatalities (Table 10). As a consequence, the mask effect is not falsely significant due to the potential success of a simultaneous testing control policy.

We also add the stringency index (*stringency*) in Tables 11 and 12 to control the existence of other control/lockdown measures during the same periods the masks have been used. Thus, we are able to disentangle the effects from the masks and the effects stemming from other control and mitigation measures. The mask use effect is negative and significant at high significance level ($P < 0.01$) for lags 14 and 28 (infected cases) and lag 14 and 42 for fatality rates regressions (at a lower significance level).

Table 1: Table S1: Fatality rate model with tests per 100k control variable as an additional control, global sample, infected cases, MG estimates

VARIABLES	(1) cases	(2) cases	(3) cases	(4) cases
cases (t-1)	0.179***	0.161***	0.121***	0.0285
	(0.039)	(0.039)	(0.039)	(0.041)
cases (t-14)	0.105***	0.115***	0.137***	0.184***
	(0.025)	(0.026)	(0.034)	(0.040)
masks (t-7)	-1.850			
	(1.520)			
new tests (t-7))	0.016			
	(0.976)			
masks(t-14)		-6.050***		
		(2.260)		
new tests (t-14)		-0.158		
		(0.655)		
masks (t-28)			-5.980***	
			(2.210)	
new tests (t-28)			0.014	
			(0.013)	
masks (t-42)				-66.600
				(1.820)
new tests (t-42)				0.010
				(0.014)
time	4.200*	4.900**	7.900***	8.370**
	(2.440)	(1.970)	(2.760)	(3.590)
Constant	-0.001*	-0.001**	-0.001***	-0.001**
	(0.001)	(0.001)	(0.001)	(0.001)
Observations	4,779	4,351	3,454	2,558
Number of id	68	68	67	66

Standard errors in parentheses. 'Cases' means cases rates.
The numbers of all variables, excepting cases, multiple e^{-8}.
*** p<0.01, ** p<0.05, * p<0.1

Table 2: Table S2: Fatality rate model with tests per 100k control variable as an additional control, global sample, infected cases, MG estimates

VARIABLES	(1) fatality rate	(2) fatality rate	(3) fatality rate
fatality rate (t-1)	0.001	-0.046**	-0.071***
	(0.027)	(0.021)	(0.025)
fatality rate (t-14)	0.011	0.018	0.048**
	(0.018)	(0.018)	(0.022)
cases (t-14)	0.001	-0.001	-0.001
	(0.001)	(0.001)	(0.001)
masks (t-14)	-1.180**		
	(53.400)		
new tests (t-14)	0.012***		
	(0.423)		
masks (t-28)		-1.080*	
		(58.800)	
new tests (t-28)		0.383	
		(0.398)	
masks (t-42)			-31.200
			(55.400)
new tests (t-42)			0.298
			(0.461)
time	-1.300	-20.100	2.710
	(8.960)	(1.070)	(1.910)
Constant	0.0001	0.004	-0.0001
	(0.0001)	(0.0002)	(0.0001)
Observations	4,351	3,454	2,558
Number of id	68	67	66

Standard errors in parentheses

The numbers of all variables, excepting fatality, multiple e^{-9}.

*** p<0.01, ** p<0.05, * p<0.1

Table 3: Table S3: Fatality rate model with tests per 100k control variable as an additional control, global sample, infected cases, MG estimates

VARIABLES	(1) cases	(2) cases	(3) cases	(4) cases
cases (t-1)	0.116***	0.107***	0.057**	0.002
	(0.027)	(0.028)	(0.025)	(0.027)
cases (t-14)	0.104***	0.117***	0.105***	0.138***
	(0.022)	(0.023)	(0.026)	(0.031)
masks (t-7)	-1.570			
	(1.430)			
stringent (t-7)	78.500			
	(1.970)			
masks (t-14)		-5.730***		
		(1.730)		
stringent (t-14)		99.300		
		(2.210)		
masks (t-28)			-4.930***	
			(1.680)	
stringent (t-28)			1.810	
			(2.820)	
masks (t-42)				-90.000
				(1.230)
stringent (t-42)				1.400
				(2.630)
time	4.410**	4.900***	7.050**	8.120*
	(1.820)	(1.860)	(2.800)	(4.250)
Constant	-0.001**	-0.001***	-0.001**	-0.001*
	(0.001)	(0.001)	(0.001)	(0.001)
Observations	7,254	6,596	5,280	3,959
Number of id	95	95	95	95

Standard errors in parentheses

The numbers of all variables, excepting cases, multiple e^{-8}.

*** p<0.01, ** p<0.05, * p<0.1

Table 4: Table S4: Fatality rate model with tests per 100k control variable as an additional control, global sample, infected cases, MG estimates

VARIABLES	(1)<br>fatality rate	(2)<br>fatality rate	(3)<br>fatality rate	(4)<br>fatality rate
fatality rate (t-1)	-0.025	-0.015	-0.040***	-0.090***
	(0.020)	(0.020)	(0.015)	(0.017)
fatality rate (t-14)	0.017	0.028*	0.030*	0.036
	(0.018)	(0.017)	(0.017)	(0.023)
cases (t-14)	0.001	0.001	-0.0003	-0.001
	(0.001)	(0.001)	(0.001)	(0.001)
masks (t-7)	-8.670*			
	(4.580)			
stringency (t-7)	-5.370			
	(8.900)			
masks (t-14)		-7.590*		
		(4.050)		
stringent (t-14)		-5.990		
		(6.230)		
masks (t-14)			-5.580	
			(4.050)	
stringency (t-28)			1.140	
			(7.650)	
masks (t-42)				-15.800**
				(7.430)
stringency (t-42)				-12.300
				(10.400)
time	-1.580	-4.110	0	-4.080
	(6.980)	(5.450)	(5.850)	(11.900)
Constant	0.001	0.001	-0.008	0.001
	(0.001)	(0.001)	(0.001)	(0.001)
Observations	7,254	6,596	5,280	3,959
Number of id	95	95	95	95

Standard errors in parentheses
The numbers of all variables, excepting fatality, multiple e^{-10}.
*** p<0.01, ** p<0.05, * p<0.1

Sample bias: considering only European, non European and Asian countries

We consider the case of European countries only (Tables 13 and 14), over the maximum time period (1st January to 15, July, 2020) using our benchmark MG estimator. Considering only European countries enables us to focus on countries with high responsiveness levels about the mask wearing variable maximizing the quality of the available information for this variable of great interest in our study. In addition, we have to take into account the fact that we have a panel of 96 countries with an important heterogeneity concerning the take-off Covid-19 periods (time with the first infected people) and so different Covid-19 dynamics over time: the first wave of Covid-19 epidemic has started later in Brazil than in Italy. Considering only homogeneous European countries is therefore a mean to test the presence of sample bias. Finally, we tested the effectiveness of masks on a set of non Eurpean countries and Asian countries only to take into account country heterogeneity (Covid waves, mask use behaviours and cultural determinants for instance).

Table 5: Table S5: Infected cases, European countries, MG estimates

	(1)	(2)	(3)	(4)
VARIABLES	cases	cases	cases	cases
cases (t-1)	0.122**	0.244***	0.226***	0.188***
	(0.051)	(0.021)	(0.021)	(0.023)
cases(t-14)	0.136***	0.257***	0.224***	0.197***
	(0.035)	(0.015)	(0.010)	(0.008)
masks (t-7)	-0.504***			
	(0.150)			
masks (t-14)		-0.112*		
		(6.290)		
masks (t-28)			-2.260	
			(9.140)	
masks (t-42)				0.272
				(0.311)
time	0.201	0.402*	0.4510	-0.669
	(0.321)	(0.229)	(0.431)	(0.016)
Constant	-0.0004	-0.0008*	-0.0009	0.0014
	(0.0007)	(0.0005)	(0.0009)	(0.003)
Observations	1,617	1,470	1,176	882
R-squared	0.100	0.183	0.113	0.071
Number of id	21	21	21	21

Standard errors in parentheses
The numbers of all variables, excepting cases, multiple e^{-9}.
*** p<0.01, ** p<0.05, * p<0.1

Table 6: Table S6: Fatalities, European countries, MG estimates

VARIABLES	(1) fatality rate	(2) fatality rate	(3) fatality rate	(4) fatality rate
fatality rate (t-1)	-0.026	0.013	0.017	-0.047
	(0.043)	(0.040)	(0.027)	(0.037)
fatality rate (t-14)	0.074*	0.062*	0.068**	0.100**
	(0.042)	(0.037)	(0.032)	(0.042)
cases (t-14)	0.002	0.002**	-0.001	-0.002*
	(0.002)	(0.001)	(0.001)	(0.001)
masks (t-7)	-4.820***			
	(1.650)			
masks (t-14)		-4.010***		
		(1.370)		
masks (t-28)			-1.700**	
			(8.550)	
masks (t-42)				-1.940
				(1.410)
time	-6.580**	-3.920**	-3.290**	-1.530
	(2.580)	(1.680)	(1.550)	(1.950)
Constant	0.001**	0.001**	0.001**	0.001
	(0.001)	(0.001)	(0.001)	(0.001)
Observations	1,617	1,470	1,176	882
Number of id	21	21	21	21

Standard errors in parentheses

The numbers of all variables, excepting fatality, multiple e^{-9}.

*** p<0.01, ** p<0.05, * p<0.1

Table 7: **Table S7: Infected cases for non European countries, MG estimates**

VARIABLES	(1)<br>cases	(2)<br>cases	(3)<br>cases	(4)<br>cases
cases (t-1)	0.159***	0.143***	0.084**	0.026
	(0.036)	(0.035)	(0.033)	(0.034)
cases (t-14)	0.103***	0.089***	0.113***	0.141***
	(0.026)	(0.026)	(0.030)	(0.037)
masks (t-7)	-2.200			
	(2.010)			
masks (t-14)		-3.890*		
		(2.170)		
masks (t-28)			-8.200***	
			(2.270)	
time	3.920*	3.980*	7.630***	0.114*
	(2.030)	(2.170)	(2.940)	(6.120)
Constant	-0.001*	-0.001*	-0.001***	-0.002*
	(0.001)	(0.001)	(0.001)	(0.001)
Observations	5,742	5,217	4,167	3,117
Number of countries	75	75	75	75

Standard errors in parentheses
The numbers of all variables, excepting cases, multiple e^{-8}.
*** p<0.01, ** p<0.05, * p<0.1

Table 8: **Table S8: Fatality rates for non European countries, MG estimates**

VARIABLES	(1)<br>fatality rate	(2)<br>fatality rate	(3)<br>fatality rate	(4)<br>fatality rate
fatality rate (t-1)	-0.004	-0.050***	-0.073***	-0.109***
	(0.023)	(0.018)	(0.020)	(0.025)
fatality rate (t-14)	0.015	0.015	0.014	0.034
	(0.019)	(0.021)	(0.027)	(0.031)
cases (t-14)	0.0003	0.0002	0.0003	0.0001
	(0.0005)	(0.0007)	(0.0005)	(0.0007)
masks (t-14)	-1.080			
	(3.570)			
masks(t-28)		-0.112**		
		(5.010)		
masks (t-42)			-2.270	
			(8.280)	
time	6.660*	8.950*	2.640	-0.197
	(3.630)	(5.230)	(0.164)	(0.136)
Constant	-0.00001*	-0.00001*	-0.00005	0.00004
	(0.0007)	(0.00001)	(0.00003)	(0.00003)
Observations	5,217	4,167	3,117	2,067
Number of countries	75	75	75	75

Standard errors in parentheses
The numbers of all variables, excepting fatality and constant, multiple e^{-9}.
*** p<0.01, ** p<0.05, * p<0.1

Table 9: Table S9: Infected cases for Asian countries only, MG estimates

VARIABLES	(1) cases	(2) cases	(3) cases	(4) cases
cases (t-1)	0.244***	0.211***	0.160**	0.072
	(0.068)	(0.064)	(0.065)	(0.067)
cases (t-14)	0.079**	0.050	0.074*	0.062
	(0.040)	(0.036)	(0.039)	(0.049)
masks (t-7)	4.780			
	(0.153)			
time	4.450	2.540	-0.228	0.231
	(0.296)	(0.401)	(0.453)	(0.967)
masks (t-14)		-7.330		
		(0.151)		
masks (t-28)			-0.299	
			(0.291)	
masks (t-42)				-0.133
				(0.177)
Constant	-0.00009	-0.00005	0.00051	-0.00050
	(0.00065)	(0.00088)	(0.00099)	(0.00213)
Observations	2,141	1,945	1,553	1,161
Number of id	28	28	28	28

Standard errors in parentheses
The numbers of all variables, excepting cases and constant, multiple e^{-9}.
*** p<0.01, ** p<0.05, * p<0.1

Taking into account additional controls: individual mobility and temperatures

We also control for mobility (driving index here). If individuals are more mobile, they are likely to use more masks to go outside (at work, at school or university, at the supermarket). Above all, the mobility can be viewed as a another proxy of the control policies (lockdown, stay-at-home requirements, restrictions on public gatherings etc): if the mobility is strongly reduced, it is an other indirect indicator that the level of stringency of the control policies is high.

We presented previous results with temperatures in the core of the manuscript (see Table 5), for 67 available countries. Considering our data about mobility and temperatures, we are working on a shorter sample of European countries here. When we add other control variables, the number of countries dropped to 22. For transparency concerns, we first present the results of the mask wearing impact before introducing additional controls: temperatures, mobility and new tests per 100k inhabitants. The mask variable is negatively associated to the cases ratio with very high level of significance ($P < 0.01$) for 7 and 14 days/lags. Then, we add other controls in this benchmark regression.

Table 10: Table S10: Benchmark regression before introducing additional controls, European countries, MG estimates

VARIABLES	(1) cases	(2) cases	(3) cases
casepop (t-1)	0.046	0.015	-0.199*
	(0.062)	(0.049)	(0.109)
casepop (t-14)	0.049	0.048	0.136*
	(0.032)	(0.042)	(0.071)
masks (t-7)	-0.009***		
	(0.002)		
masks (t-14)		-0.006***	
		(0.002)	
masks (t-28)			-0.003
			(0.002)
Constant	1.664***	1.452***	0.816***
	(0.300)	(0.290)	(0.204)
Observations	616	462	154
Number of countries	22	22	22

Standard errors in parentheses
*** p<0.01, ** p<0.05, * p<0.1

Table 11: Table S11: Infected cases with tests per 100k control variable (lagged 7 days), European countries only, MG estimates

VARIABLES	(1) cases	(2) cases
cases (t-1)	0.007	-0.270*
	(0.059)	(0.144)
cases (t-14)	0.041	0.172*
	(0.033)	(0.098)
masks (t-7)	-0.009**	
	(0.004)	
new tests number (t-7)	-0.0001	
	(0.0001)	
masks (t-28)		-0.002
		(0.009)
new tests number (t-28)		-0.0002
		(0.001)
Constant	1.423***	1.165
	(0.317)	(0.833)
Observations	616	154
Number of countries	22	22

Standard errors in parentheses
*** p<0.01, ** p<0.05, * p<0.1

Table 12: **Table S12: Infected cases with temperatures and mobility/driving variables, European countries only, MG estimates**

VARIABLES	(1)<br>cases	(2)<br>cases	(3)<br>cases
cases (t-1)	-0.026	-0.052	-0.070
	(0.058)	(0.059)	(0.058)
cases (t-14)	0.040	0.018	0.009
	(0.029)	(0.032)	(0.027)
masks (t-7)	-0.010**	-0.010*	-0.010
	(0.005)	(0.005)	(0.007)
new tests number (t-7)	-0.0001	-0.0003	-0.0001
	(0.0001)	(0.0002)	(0.0002)
temperature (t-7)	-0.024	-0.002	0.009
	(0.102)	(0.153)	(0.156)
mobility (t-7)		-0.004	-0.005
		(0.003)	(0.009)
mobility × mask (t-7)			-0.0001
			(0.0001)
Constant	1.401***	1.125*	1.275
	(0.379)	(0.597)	(0.819)
Observations	616	572	572
Number of countries	22	22	22

Standard errors in parentheses.
Mobility is 'driving' from Google Mobility reports.
*** p<0.01, ** p<0.05, * p<0.1

3. Determinants of mask use: additional statistical analysis

3.1. Matrix of correlation and potential collinearity

We have checked the complete matrix of correlations to avocountries collinearity problems. For example, rich countries characterized by higher GDP levels (in natural logarithms units) have a high probability to have also a high proportion of elder people and high government effectiveness index. As a consequence, a mutlivariable regression with GDP, $Age65$ and gov_effect simultaneously is not robust due to the presence of collinearity issues.

Table 13: Table S13: Cross-correlation table

Variables	density	co2	stringency	gdp	age652017	gov_effe	cum cases	altruism
density	1.000							
co2	0.094	1.000						
stringency	-0.140	0.029	1.000					
gdp	0.116	0.478	-0.347	1.000				
age652017	0.082	0.326	-0.597	0.675	1.000			
gov_effectiveness_2018	0.174	0.334	-0.500	0.835	0.715	1.000		
cum cases	0.110	0.148	0.217	0.424	-0.038	0.234	1.000	
altruism	0.048	-0.195	0.136	-0.287	-0.252	-0.182	-0.041	1.000

3.2. Partial linear regressions

In our cross-sectional database, we select mask use proportion variable on July, 15, 2020 corresponding to the latest observation of our daily frequency panel dataset. We also select the mask use proportion variable in the beginning period (this date varies from April, 23 to April, 25, 2020 according to the countries) to take into account the dynamics of the pandemic and the potential adjustments in human behaviors regarding the mask wearing as well as the effects of the control policies implemented by the governments.

We have tested partial linear regressions for all potential determinants. We have considered the presence of potential quadratic forms even though our cross-section sample is of a limited size. In the following tables, we present the most suitable specifications and only report the most significant regression: for instance, population density regression does not incorporate significant quadratic variable whereas CO2 emissions influence on mask use is working via a quadratic form; in other words, only highly polluted countries are characterized by a positive influence of pollution level (level of CO2 emissions in natural logarithm) on the percentage of population wearing a face mask.

These partial regressions give information about the potential socioeconomic determinants but need to be cautiously analysed due to the presence of potential issues, especially omitted variable bias and serial correlation issues.

3.3. Partial linear regressions: observations on July, 15, 2020

Table 14: Table S14: Masks determinants on July, 15, 2020: partial linear regressions (part A)

VARIABLES	(1) masks	(2) masks	(3) masks	(4) masks	(5) masks	(6) masks
co2 (log)		-0.324***				
		(0.107)				
$co2^2 (log)$		0.015***				
		(0.005)				
density (log)	0.043**					
	(0.019)					
stringency			0.004***			
			(0.001)			
diabete				0.065***		
				(0.024)		
$diabete^2$				-0.002**		
				(0.001)		
lgdp17					-0.036	
					(0.028)	
age652017						-0.009**
						(0.004)
Constant	0.395***	2.207***	0.298***	0.259**	0.932***	0.681***
	(0.094)	(0.546)	(0.089)	(0.126)	(0.268)	(0.041)
Observations	89	89	87	90	89	90
R-squared	0.056	0.073	0.150	0.065	0.021	0.072

Robust standard errors in parentheses
*** p<0.01, ** p<0.05, * p<0.1

Table 15: Table S15: Masks determinants on July, 15, 2020: partial linear regressions (part B)

VARIABLES	(1) masksfinal	(2) masksfinal	(3) masksfinal	(4) masksfinal	(5) masksfinal	(6) masksfinal
gov_effectiveness_2018	-0.057					
	(0.035)					
pct_overweight		-0.003**				
		(0.001)				
altruism			0.052			
			(0.081)			
tolerance				0.052***		
				(0.016)		
$tolerance^2$				-0.001***		
				(0.001)		
cum cases0707					8.301**	
					(3.502)	
cum cases0906						7.499
						(4.513)
Constant	0.591***	0.755***	0.594***	-0.796	0.556***	0.566***
	(0.023)	(0.070)	(0.029)	(0.527)	(0.029)	(0.028)
Observations	87	86	59	52	88	88
R-squared	0.054	0.047	0.007	0.380	0.027	0.010

Robust standard errors in parentheses
*** p<0.01, ** p<0.05, * p<0.1

3.4. Partial linear regressions: observations on April, 23-25, 2020

Table 16: Table S16: Masks determinants on April, 23-25, 2020: partial linear regressions (part B)

	(1)	(2)	(3)	(4)	(5)	(6)
VARIABLES	masks	masks	masks	masks	masks	masks
co2 (log)		-6.345				
		(5.474)				
co2$^2(log)$		0.419				
		(0.269)				
density (log)	0.754					
	(0.969)					
stringency			0.150**			
			(0.0728)			
diabete				1.752		
				(1.249)		
diabete^2				-0.087*		
				(0.047)		
gdp (log)					-0.578	
					(1.150)	
age65						0.092
						(0.215)
Constant	14.830***	36.08	8.960**	10.730	23.520**	17.030***
	(4.701)	(27.05)	(4.315)	(6.458)	(10.97)	(2.387)
Observations	89	89	87	90	89	90
R-squared	0.005	0.120	0.052	0.019	0.002	0.002

Robust standard errors in parentheses
*** p<0.01, ** p<0.05, * p<0.1

Table 17: Table S17: Masks determinants on April, 23-25, 2020: partial linear regressions (part B)

VARIABLES	(1)<br>masks	(2)<br>masks	(3)<br>masks	(4)<br>masks
gov_effectiveness_2018	-1.317			
	(1.315)			
pct_overweight		-0.038		
		(0.078)		
altruism			-7.081	
			(5.463)	
tolerance				2.061*
				(1.219)
$tolerance^2$				-0.018**
				(0.009)
Constant	18.660***	20.480***	20.290***	-29.760
	(1.482)	(3.944)	(1.943)	(39.190)
Observations	87	86	59	52
R-squared	0.009	0.002	0.027	0.188

Robust standard errors in parentheses
*** p<0.01, ** p<0.05, * p<0.1

3.5. Effect of education: observations on July, 15, 2020

We have tested the effect of education using the last available data about the schooling variable from the World Bank and also the last PISA score. We expect that a better education level enhance the mask wearing as well as hygiene and compliance in a general way.

Table 18: Table S18: Education effect estimates

VARIABLES	(1) masks	(2) masks
log(density_2017)	0.036**	0.091***
	(0.018)	(0.021)
log(co2_2014)	-0.150	-0.953*
	(0.156)	(0.506)
$\log(co2\_2014)^2$	0.007	0.042*
	(0.006)	(0.021)
cumulated_cases0707 pop100	0.018	0.074
	(0.023)	(0.107)
stringency	0.006***	0.006*
	(0.001)	(0.003)
school_2017	-0.003	
	(0.003)	
PISA		-0.001
		(0.001)
Constant	1.079	5.542*
	(0.721)	(3.095)
Observations	74	33
R-squared	0.368	0.524

Robust standard errors in parentheses
*** p<0.01, ** p<0.05, * p<0.1

3.6. Effect of overweight population: observations on July, 15, 2020

We have tested the effect of overweight using the last available data about the overweight proportion variable.

Table 19: Table S19: Overweight effect estimates

VARIABLES	(1) masks	(2) masks
density		0.027
		(0.021)
co2		-0.304***
		(0.101)
$co2^2$		0.014***
		(0.004)
cum_cases0707		0.102**
		(0.040)
diabete		0.009
		(0.006)
pct_overweight	0.019*	0.028***
	(0.010)	(0.009)
pct_overweight^2	-0.001**	-0.001***
	(0.0001)	(0.0001)
Constant	0.323	1.437**
	(0.206)	(0.546)
Observations	86	83
R-squared	0.084	0.244

Robust standard errors in parentheses
** 4* p<0.01, ** p<0.05, * p<0.1

3.7. Additional regressions: controlling for non-pharmaceutical measures

We control for the effect of other mitigation measures on mask wearing on July, 15, 2020 by introducing four dummy variables in our regression model: travel restriction (*travel*), testing policy (*testing*), school closures policy (*school*), surveillance and tracking policy (*surveillance*). All data come from the Porcher (2020) database at https://response2covid19.org/.

Table 20: Table S20: Estimates with mitigation policies controls

VARIABLES	(1) masks	(2) masks	(3) masks
density		0.043**	0.045**
		(0.018)	(0.018)
co2		-0.365***	-0.365***
		(0.111)	(0.113)
$co2^2$		0.017***	0.017***
		(0.005)	(0.005)
stringency		0.004***	0.005***
		(0.001)	(0.001)
cum cases0707			0.028
			(0.026)
testing	-0.011	0.057	0.081
	(0.067)	(0.055)	(0.055)
surveillance	-0.023	0.023	0.015
	(0.090)	(0.063)	(0.062)
school	-0.242	-0.188	-0.176
	(0.210)	(0.122)	(0.123)
travel	0.102	0.022	0.034
	(0.095)	(0.080)	(0.082)
Constant	0.507***	1.947***	1.900***
	(0.091)	(0.553)	(0.558)
Observations	89	84	82
R-squared	0.037	0.333	0.363

Robust standard errors in parentheses
*** p<0.01, ** p<0.05, * p<0.1

3.8. Additional regressions: controlling for trust in government

Some studies assume that differences in government and politicians trust can impact the effectiveness of control policies (see for instance Bargain and Aminijonov, 2020). We computed the proportion of people that have 'not trust at all' in their government from World Value Survey database (2017-2020 survey) as a "trust in government" proxy. A high proportion of people that do not trust in government is not associated to a reduction of mask wearing proportion (see figure 9). However, note that the number of observation is limited and results need to be cautiously interpreted. We can not completely confirm that people that do not trust at all are the mask offenders and explain heterogeneity about mask wearing across countries.

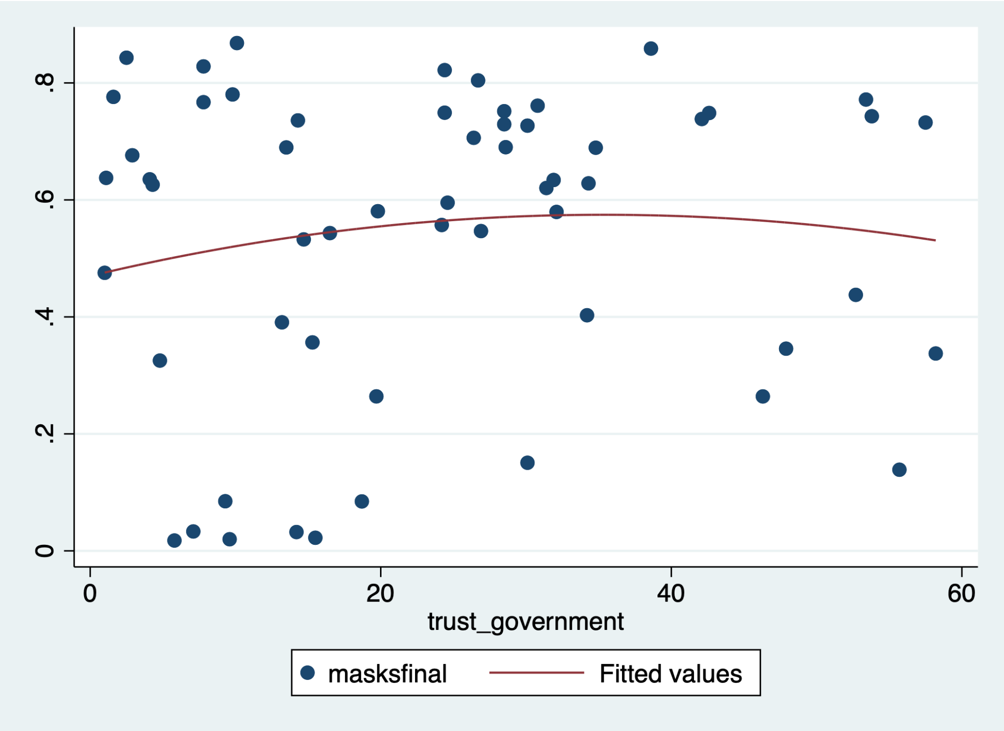

Figure 1: Mask adoption versus trust in government index

Note: The X-axis refers to the mask adoption proportion: a 0.8 value means that 80 respondents over 100 declared to use a face mask. The Y-axis denotes the trust in government index (a large value indicates that a high proportion of people are not confident at all in their government. 'maskfinal' denotes the proportion of people using a face mask in the final observation of our sample (15th July, 2020). The red line refers to a simple linear fit.

Table 21: Table S21: Estimates with trust in government variable

VARIABLES	(1)<br>masksfinal	(2)<br>masksfinal
density	0.051*	0.064*
	(0.026)	(0.035)
co2014	-0.085	-0.119
	(0.406)	(0.419)
co2sq	0.006	0.008
	(0.017)	(0.017)
stringency	0.005***	0.005***
	(0.001)	(0.001)
cum cases0707		0.040
		(0.101)
government_confidence	0.002	0.002
	(0.001)	(0.001)
Constant	-0.0001	0.143
	(2.384)	(2.446)
Observations	45	44
R-squared	0.416	0.422

Robust standard errors in parentheses
*** p<0.01, ** p<0.05, * p<0.1

4. Descriptive Statistics

Table 22: Table S22: Descriptive Statistics for the panel dataset

	Sum	Mean	SD	Min	Max	N
casepop	0.30050	1.541-05	4.732E-05	0.0000	0.00190	19503
deathpop	0.01013	5.198E-07	1.843E-06	0.0000	0.00004	19503
masks	4581.11	0.56466	0.23427	0.00889	0.91000	8113
masksN	711887.1	87.7464	78.8736	1.1777	477.970	8113
mask_dummy	6600.00	0.35000	0.48000	0.0000	1.000	18909
temperature	359155.0	17.2306	9.0276	-14.107	38.3699	20844
stringency	954159.7	54.8525	32.7465	0.0000	100.00	17395
new_tests_per_thousand	3943.742	0.45868	0.69150	-0.212	7285.0	8598

Table 23: Table S23: Cross-correlation table

Variables	casepop	deathpop	masksN	mask_dummy	temperature	stringency	new_tests
casepop	1.000						
deathpop	0.378	1.000					
masksN	0.045	0.158	1.000				
mask_dummy	0.212	0.019	0.128	1.000			
temperature	0.107	-0.082	-0.048	0.345	1.000		
stringent	0.241	0.212	0.002	0.376	0.330	1.000	
new_tests	0.281	0.116	-0.145	0.042	-0.052	0.029	1.000

5. List of countries

Considering available data, 96 countries have been finally considered in our statistical analysis:

Afghanistan Albania Algeria Angola Argentina Armenia Australia Austria Azerbaijan Bangladesh Belarus Belgium Bolivia Bosnia and Herzegovina Brazil Bulgaria Burkina Faso Cambodia Cameroon Canada Chile Colombia Costa Rica Croatia Cyprus Czechia Denmark Dominican Republic Ecuador Egypt Salvador Estonia Ethiopia Finland France Georgia Germany Ghana Greece Guatemala Honduras Hong Kong Hungary Iceland India Indonesia Iran Iraq Ireland Israel Italy Jamaica Japan Jordan Kazakhstan Kenya Kuwait Kyrgyzstan Laos Libya Lithuania Madagascar Malaysia Mali Mexico Moldova Morocco Nepal Netherlands New Zealand Nicaragua Nigeria Norway Oman Pakistan Panama Paraguay Peru Philippines Poland Portugal Qatar Republic of Korea Romania Russia Saudi Arabia Serbia Singapore Slovakia Slovenia South Africa Spain Sri Lanka Sudan Sweden Switzerland Taiwan Tanzania Thailand Tunisia Turkey Ukraine United Arab Emirates United Kingdom United States of America Uruguay Uzbekistan Venezuela Vietnam

Table 24: Table S24: Masks effects on Covid-19 infected cases with an alternative mask use proxy

VARIABLES	(1)<br>cases rate	(2)<br>cases rate	(3)<br>cases rate
cases rate (t-1)	0.358***	0.335***	0.254***
	(0.041)	(0.044)	(0.046)
cases rate (t-14)	0.091***	0.102***	0.083***
	(0.023)	(0.026)	(0.025)
mask_dummy (t-14)		-30.400**	
		(12.600)	
temperature (t-14)		-3.210	
		(4.270)	
stringency index (t-14)		-1.490	
		(1.460)	
new test number (t-14)		103.000	
		(111.000)	
mask_dummy (t-28)			-4.540
			(3.700)
temperature (t-28)			-2.170
			(4.760)
stringency index (t-28)			-3.500**
			(1.580)
news tests number (t-28)			-257.000**
			(131.000)
time	15.300	1.210	1.180
	(1.360)	(1.790)	(2.050)
Constant	-349.000	-0.001	-0.001
	(0.001)	(0.001)	(0.001)
Observations	7,467	7,032	6,115
Number of countries	70	70	70

Standard errors in parentheses
*** p<0.01, ** p<0.05, * p<0.1

6. Alternative measure of face mask use: estimates with a binary variable
